# Predicted overlapping microRNA regulators of acetylcholine packaging and degradation in neuroinflammation-related disorders

**DOI:** 10.3389/fnmol.2014.00009

**Published:** 2014-02-10

**Authors:** Bettina Nadorp, Hermona Soreq

**Affiliations:** Department of Biological Chemistry and the Center for Bioengineering, The Edmond and Lily Safra Center for Brain Science, The Hebrew University of JerusalemJerusalem, Israel

**Keywords:** acetlycholinesterase, butyrylcholinesterase, choline acetyltransferase, vesicular acetylcholine transporter, microRNA-186, cholinergic signaling, primate-specific microRNAs

## Abstract

MicroRNAs (miRNAs) can notably control many targets each and regulate entire cellular pathways, but whether miRNAs can regulate complete neurotransmission processes is largely unknown. Here, we report that miRNAs with complementary sequence motifs to the key genes involved in acetylcholine (ACh) synthesis and/or packaging show massive overlap with those regulating ACh degradation. To address this topic, we first searched for miRNAs that could target the 3′-untranslated regions of the choline acetyltransferase (ChAT) gene that controls ACh synthesis; the vesicular ACh transporter (VAChT), encoded from an intron in the ChAT gene and the ACh hydrolyzing genes acetyl- and/or butyrylcholinesterase (AChE, BChE). Intriguingly, we found that many of the miRNAs targeting these genes are primate-specific, and that changes in their levels associate with inflammation, anxiety, brain damage, cardiac, neurodegenerative, or pain-related syndromes. To validate the *in vivo* relevance of this dual interaction, we selected the evolutionarily conserved miR-186, which targets both the stress-inducible soluble “readthrough” variant AChE-R and the major peripheral cholinesterase BChE. We exposed mice to predator scent stress and searched for potential associations between consequent changes in their miR-186, AChE-R, and BChE levels. Both intestinal miR-186 as well as BChE and AChE-R activities were conspicuously elevated 1 week post-exposure, highlighting the previously unknown involvement of miR-186 and BChE in psychological stress responses. Overlapping miRNA regulation emerges from our findings as a recently evolved surveillance mechanism over cholinergic neurotransmission in health and disease; and the corresponding miRNA details and disease relevance may serve as a useful resource for studying the molecular mechanisms underlying this surveillance.

## Introduction

MicroRNAs (miRNAs) are small, 20–25 nucleotides long, non-coding RNA molecules, each of which can predictably target many protein-coding messenger RNA (mRNA) transcripts to silence them post-transcriptionally. Mammalian miRNAs bind target mRNAs via a short “seed” sequence, such that many miRNAs can target the same mRNAs, and different mRNAs may be targeted by a single miRNA gene (Bartel, 2009). Interestingly, miRNAs often target different mRNAs all involved in a particular biological function (Chen et al., 2004). This has been extensively studied in various cancers (Kefas et al., 2008; Papagiannakopoulos et al., 2008; Levy et al., 2010; Lupini et al., 2013), but the control by miRNAs of specific neurotransmission processes remained largely unexplored. In principle, one would predict that the synthesis, packaging in neuronal vesicles and destruction or re-uptake of a certain neurotransmitter should be co-regulated; this, in turn implies that some miRNAs may co-suppress two or more of the mRNA transcripts involved in regulating the levels of certain neurotransmitters, and that modified expression of such miRNAs might be involved in diseases associated with impaired regulation of this neurotransmission pathway. Based on this working hypothesis, we studied miRNA-mediated regulation of mRNA transcripts involved in the synthesis, vesicle packaging, and destruction of acetylcholine (ACh).

**Figure 1:**
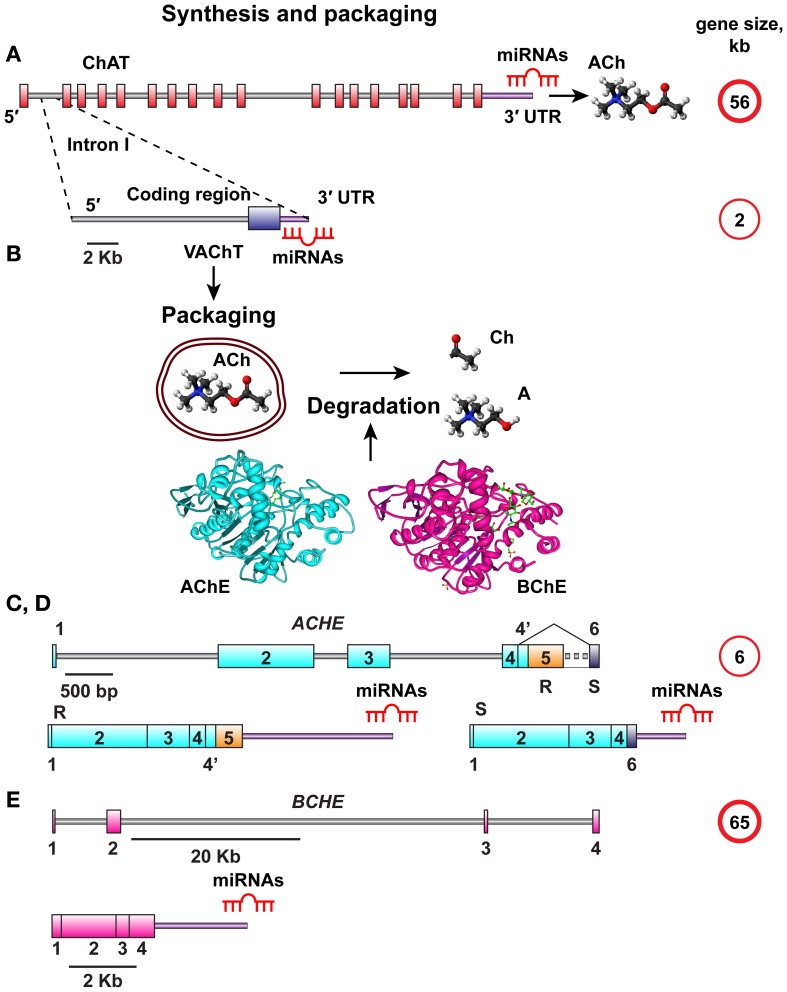
**Schematic overview of ACh synthesis, packaging, and destruction. (A)** The choline acetyl transferase (ChAT) gene responsible for ACh synthesis is 56 kb in size and all its splice variants have the same 3′UTR. **(B)** The vesicular acetylcholine transporter (VAChT) controlling ACh packaging into vesicles is encoded from the first intron of the ChAT gene. The VAChT gene is only 2 kb in length, yet it has its own 3′UTR. Once released, ACh should be degraded into Acetate and Choline by one of the splice variants of AChE or BChE, depending on its site of release. **(C,D)** The AChE gene is 6 kb in size, and its mRNA transcript is spliced to yield the major AChE-S and AChE-R splice variants with distinct 3′-UTR domains. **(E)** The BChE gene is 65 kb in size and yields only one known splice variant.

MiRNA-binding sequence motifs are primarily located at the 3′-untranslated region (3′-UTR) of the mRNA transcript (Bartel, 2009). Therefore, we interrogated the 3′-UTR domains in the choline acetyltransferase (ChAT) gene, which is responsible for ACh synthesis and the vesicular acetylcholine transporter (VAChT), encoded from the first intron in the ChAT gene (Erickson et al., 1994; Eiden, 1998). The VAChT transcript has its own 3′-UTR, which might suggest that it can be regulated by distinct miRNAs. Given that released ACh is degraded by the ACh hydrolyzing enzymes acetyl- and butyrylcholinesterase (AChE, BChE) (Meshorer and Soreq, 2006), and since increased AChE synthesis may be linked with decreased ChAT production (Kaufer et al., 1998), we further searched for potential overlaps in the predicted miRNAs between the human ChAT/VAChT 3′-UTRs and the alternative 3′-UTR choices of the major variants of AChE mRNA, the “synaptic” AChE-S and the “read-through” variant AChE-R which shares its 3′-UTR with the “erythrocytic” AChE-E isoform and is known to be induced under stress (Meshorer and Soreq, 2006). Of note, the AChE-S variant possesses a considerably shorter 3′-UTR compared to AChE-R (Hanin and Soreq, 2011) with further likelihood of differential miRNA regulation for the AChE-R and AChE-S targets. The BChE gene, completing the series of regions to be analyzed, has a different 3′-UTR with lower G, C-content (Soreq and Seidman, 2001), demonstrating sequence differences. All of these regions are functionally involved in cholinergic signaling, and we therefore designated the corresponding miRNAs “CholinomiRs.” A schematic overview of the transcripts involved in this pathway is given in Figure 1.

There were only a few overlaps between the predicted CholinomiRs regulating the synthesis and destruction of ACh. In contrast, we identified numerous overlaps between those CholinomiRs controlling ACh packaging and its synthesis; suggesting that miRNAs play an important role in selectively co-regulating cholinergic signaling by adapting the rates and efficacy of ACh packaging and destruction. Changes in these CholinomiRs, of which many are primate-specific, were further reported by others in inflammation and anxiety, brain damage, pain, cardiac, and neurodegenerative diseases, all of which are known for cholinergic signaling impairments.

To test the relevance of our predictions for *in vivo* conditions, we subjected mice to the long-lasting predator scent stress (Zimmerman et al., 2012) and tested, 1 week later, for changes in one miRNA, miR-186 and its predicted targets AChE-R and BChE. Given our previous findings of miRNA regulation of cholinergic-mediated production of intestinal miR-132 (Shaked et al., 2009), we quantified miR-186 levels in intestinal sections and measured cholinesterase activities. We found that predator scent stress induces intestinal increases in both the cholinesterases-targeting miR-186 and in the activities of the targeted cholinesterases. Our findings support the hypothesis that overlapping CholinomiR regulation serves as a recently evolved surveillance mechanism that can balance cholinergic signaling in brain and peripheral systems. The detailed lists of these miRNAs and their potential involvements with different diseases may be a valuable resource for researchers interested in both basic and translational aspects of key neuroinflammation and pain-related disorders.

## Materials and methods

### Bioinformatics approaches

3′-UTR sequences of the human ChAT, VAChT, AChE-S, AChE-R, and BChE transcripts were acquired from the NCBI nucleotide database (Entrez Nucleotide, 2010 http://www.ncbi.nlm.nih.gov/nuccore/). These sequences are 380, 58, 219, 963, and 477 nucleotides long, respectively. MiRNA-mRNA interactions were addressed by using four different algorithms, miRBase (Last update in June 2013, http://www.mirbase.org/), TargetScan (Last update in June 2012, http://www.targetscan.org/vert_50/), microcosm (version 5) (http://www.ebi.ac.uk/enright-srv/microcosm/htdocs/targets/v5/) and miRanda (Last update in August 2010, http://www.microrna.org/microrna/home.do) which were last updated in June 2013, June 2012 and August 2010, respectively. All predictions ensured a threshold *P*-value < 0.05, and analysis specifications allowed both evolutionarily conserved and non-conserved miRNAs, which further enabled us to differentiate between primate-specific and evolutionarily conserved miRNAs.

To gain more information on the identified miRNAs and assess their prospects to interact with their targets, we determined the G, C content of all identified miRNAs using G, C content calculator algorithms (http://www.endmemo.com/bio/gc.php). We further focused on the overlapping miRNAs targeting more than one 3′-UTR of the 5 studied transcripts and distinguished primate-specific from evolutionarily conserved miRNAs that appeared in our list using the HomoloGen conservation score^1^ that reports genomic conservation values between tested genes from different species. For data-mining regarding the relation to specific diseases we utilized PubMed and Google Scholar.

## Experimental *in vivo* tests

To experimentally test the putative association between changes in the identified miRNAs and their protein targets under stressful conditions, we exposed C57/B6J mice to predator scent and additionally injected them for four consecutive days with 50 μ g kg^−1^ saline, essentially as in (Zimmerman et al., 2012). 7 days later, we removed intestinal sections from these mice and matched male control mice, as in (Shaked et al., 2009). The mice were housed four per cage, at 21 ± 1°C, in a 12-h light/dark cycle. The RNA extraction procedure followed that of (Maharshak et al., 2013) for human intestinal biopsies. Extracted RNA from the intestinal sections was used to quantify miR-186 and RNU6 levels by qRT-PCR (PerfeCTa® microRNA Assay). RNU6 snRNA levels were used to normalize the levels of miR-186. The following primers were employed: RNU6 (Quanta, Cat. # HS-RNU6), miR-186-5p (Quanta, Cat. # HSMIR-0186-5p), PerfeCTa® Universal PCR Primer (Quanta, Cat. # 95109-500). Protein extraction from the intestinal sections was performed by using Solution D (0.01 M TRIS, 1 M NaCl, 1 mM EGTA, 1% T-X100). Cholinesterase activities in the tissue homogenates were determined by quantifying acetylthiocholine hydrolysis rates (Ellman et al., 1961) in the presence or absence of iso-OMPA to selectively block BChE activities, all as under (Arbel et al., 2014). Animal procedures followed the ethics instructions at The Hebrew University of Jerusalem (Ethics number of research: NS-10205-4).

## Statistical analysis

Collected data was summarized and displayed as mean ± standard deviation (Erickson et al., 1994) using SPSS software (version 19.0, SPSS INC, Chicago, IL, USA). Normally distributed variables were compared using Student's *t* test. The level of significance used for all of the above analyses was 2-tailed *p* < 0.05.

## Results

3′-UTR sequences of the human ChAT, VAChT, AChE-S, AChE-R, and BChE transcripts were acquired from the NCBI nucleotide database. These sequences span 380, 58, 219, 963, and 477 nucleotides, respectively and differ in their G, C content. We identified 42, 67, 55, 125, and 205 complementary miRNAs predicted to bind to the interrogated 3′-UTRs, respectively (Table 1, for details see Supplementary Table 1). Thus, the density of miRNA binding sites in these transcripts did not simply reflect their length, and the AChE-R and BChE mRNA transcripts encoding for soluble, secreted proteins emerged as particularly susceptible for miRNA targeting. Interestingly, we found no overlap between the VAChT and ChAT targeting miRNAs, suggesting differential regulation of the ACh synthesis and packaging processes in spite of these transcripts being subject to joint transcriptional control. In contradistinction, 26% of the ChAT targeting miRNAs are also predicted to target cholinesterases, and ACh packaging and degradation seemed to share yet more miRNAs: of 67 VAChT-targeting miRNAs, 55% predictably recognize binding sites in cholinesterases as well, and 5 of them co-target both the alternatively spliced “synaptic” AChE-S and the stress and inflammation-inducible “read-through” AChE-R transcript, enabling more profound suppression of ACh hydrolysis (for details see Supplementary Table 2). Notable examples involve the conserved neurodevelopment-associated hsa-miR-125b (Martino et al., 2009) and the primate-specific hsa-miR-608 and -765 (primate specificity determined by HomoloGen conservation score^1^).

**Table 1:** **Overlapping miRs are largely primate-specific**.

**Targeted transcripts**	**Length of 3′UTR [nt]**	**G, C-content of 3′UTR**	**No of predicted miRNAs**	**G, C-content of targeting miRNAs [%]**	**% of primate specific miRNAs**
ChAT	58	59	42	57	
VAChT	380	62	67	58	
AChE-S	219	66	55	59	
AChE-R	963	62	125	55	
BChE	477	28	205	45	
VAChT + AChE-S			10	58	40
VAChT + AChE-R			24	61	54
VAChT + BChE			3	41	33
VAChT + ChAT			0	0	0
AChE-S + AChE-R			23	61	37
AChE-S + BChE			1	59	0
AChE-S + ChAT			1	82	0
AChE-R + BChE			16	46	44
AChE-R + ChAT			4	62	25
BChE + ChAT			6	52	66
AChE-S + AChE-R + VAChT			6		50
AChE-S + AChE-R + ChAT			1		100
AChE-S + BChE + VAChT			1		0

*Shown are the 3^′^-UTR lengths, G, C-content and the numbers of miRNAs predicted to target each of the ChAT, VAChT, AChE-S, AChE-R, and BChE transcripts alone and those predictably targeting more than one of these transcripts and the fractions of these miRNAs that are primate-specific*.

Our working hypothesis predicted that miRNAs targeting more than one of the five 3′-UTR domains would be more likely than others to be causally involved in regulating this entire pathway. Also, many believe that recent evolutionary processes re-shaped the miRNA landscape in primates, contributing to human higher brain functions (Khaitovich et al., 2006). To find out if this re-shaping process affected the regulation of the cholinergic system, we searched for primate specificity within the group of 76 identified miRNAs that target more than one cholinergic transcript (Supplementary Table 3). About half (49%) of the VAChT and cholinesterases co-targeting miRNAs were found to be primate-specific (Supplementary Table 3), suggesting recently evolved miRNA-mediated mechanisms for co-regulation of ACh packaging and degradation (Table 1).

Of the cholinesterase targeting miRNAs, we found 16 that predictably target both BChE and AChE-R, whereas 23 miRNAs show “seed” complementarity to both AChE-R and AChE-S. However, BChE, and the synaptic AChE-S variant share only one single miRNA (Table 1), hsa-miR-491-5p (further details on shared miRNAs can be found in Supplementary Table 2). BChE has a much lower G, C content than the other four transcripts (Soreq et al., 1990); therefore, we wanted to find out whether this is reflected in its putatively targeting miRNAs. Not surprisingly, we found the average G, C content of BChE-targeting miRNAs to be 45%, approximately 12% lower than the average G, C content of the miRNAs targeting the other four transcripts (Table 1, Supplementary Table 4).

Next, we searched for the relevance of miRNAs targeting more than one transcript involved in ACh metabolism to known disease phenotypes (Supplementary Table 5). Compatible with the cancer-associated bias in the miRNA field, we found numerous studies on cancer-related miRNAs that target more than one transcript involved in ACh metabolism (Supplementary Table 5), and yet more are likely to accumulate in the near future. However, after excluding all of these cancer-related publications, we revealed 28 out of the identified 76 miRNAs that associate with other diseases. These could be classified into five groups: inflammation and anxiety, brain damage, cardiac or neurodegenerative diseases and pain-related syndromes (Supplementary Table 6). Table 2 lists these 28 miRNAs including the transcripts they are targeting as well as their reported disease associations. Interestingly, 67% of these miRNAs play key roles in inflammation-associated diseases and 61% of them target more than one disease group.

**Table 2:**
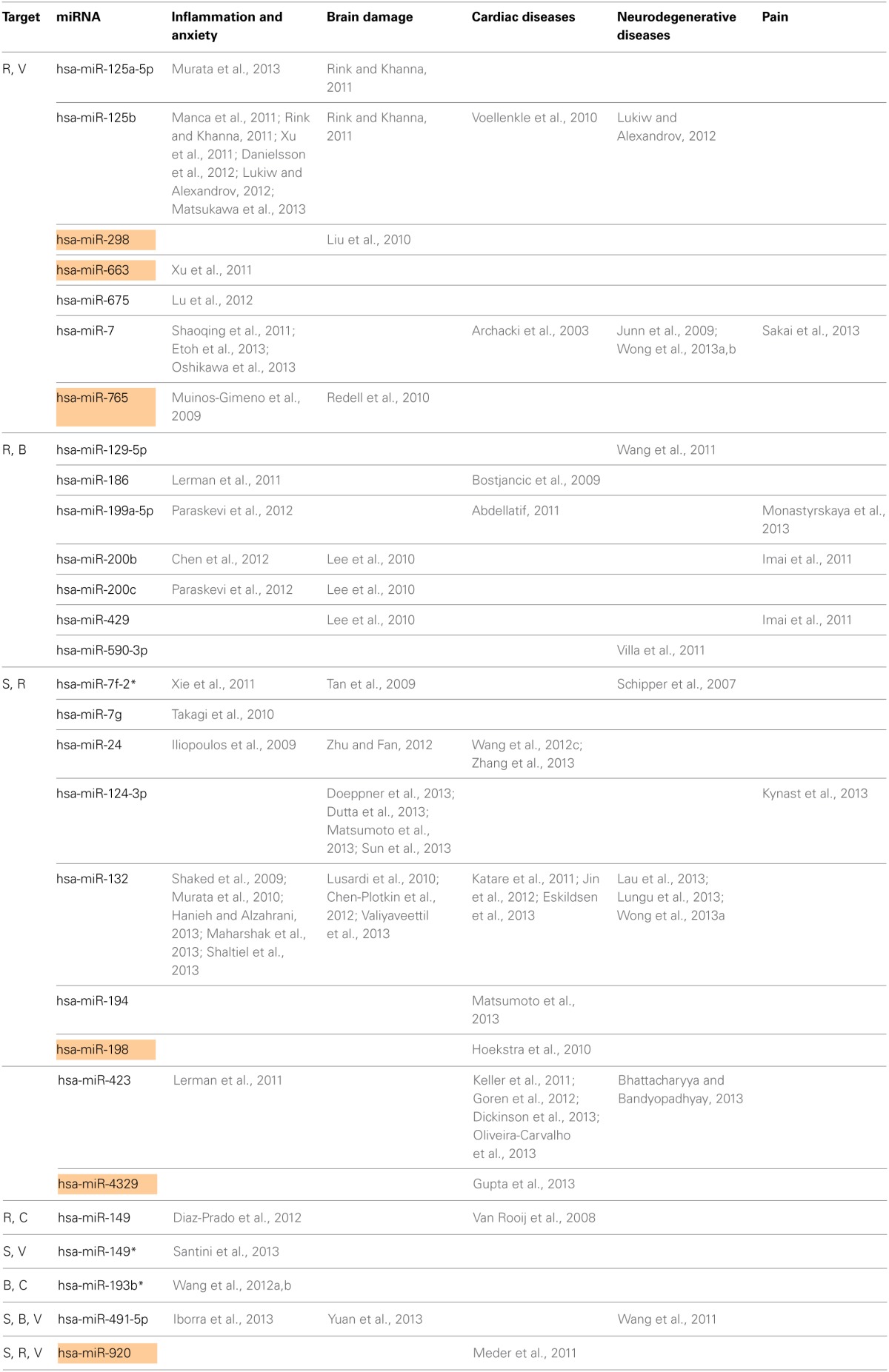
**Disease association of predicted CholinomiRs**.

*Shown are miRNAs sorted by their putative targets and the relevant reports on their disease group association. Primate specific miRNAs are marked in orange. The abbreviations C, V, S, R, and B stand for ChAT, VAChT, AChE-S, AChE-R, and BChE, respectively*.

**Figure 2:**
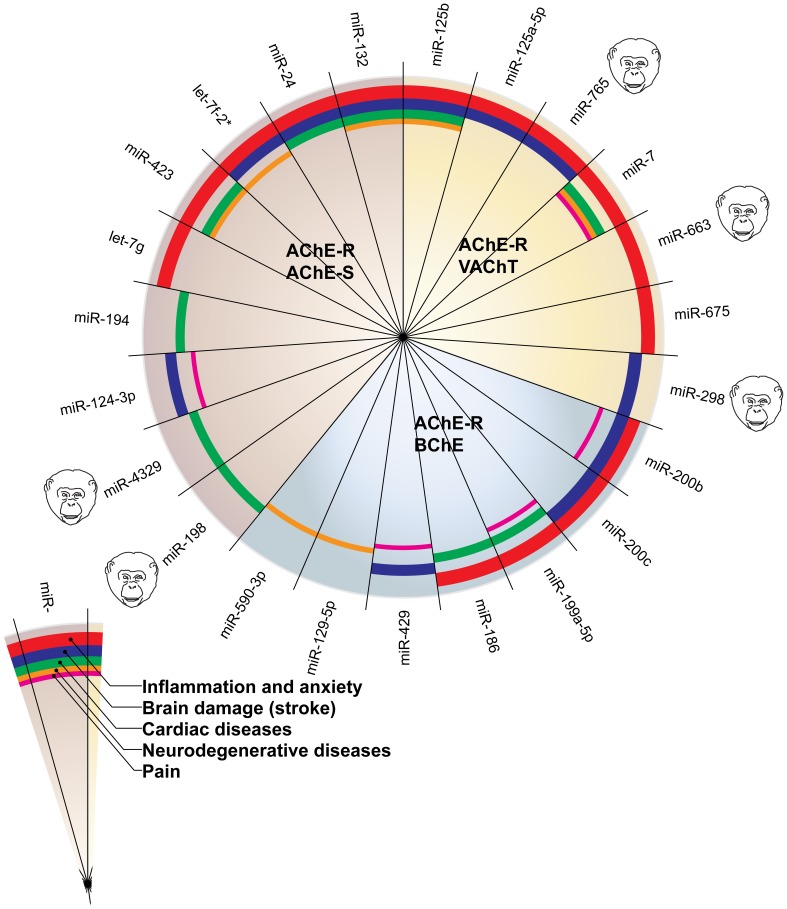
**MiRNAs targeting AChE-S and AChE-R, BChE and AChE-R or VAChT and AChE-R show associations to different disease groups related to cholinergic signaling**. The pie chart classifies the shared targets, as well as the relevant diseases and highlights the observed interactions between them. Disease group association is shown as surrounding lines of differential thicknesses, reflecting the number of miRNAs in each group. The red, blue, green, orange and purple colored lines symbolize disease associations with the groups of “inflammation and anxiety,” “brain damage (e.g., stroke),” “cardiac diseases,” “neurodegenerative diseases,” and “pain,” respectively. Note that miR-132 and miR-125b associate with all disease groups. The monkey heads next to specific miRNA numbers symbolize primate specificity.

Figure 2 presents the miRNAs co-targeting AChE-S and AChE-R, BChE, and AChE-R or VAChT and AChE-R in a pie chart classifying the shared targets and relevant diseases and highlighting the observed interactions between these miRNAs and the disease phenotypes, as well as the primate specificity of part of these miRNAs. Disease group association is shown as surrounding lines of differential thicknesses, reflecting the number of miRNAs in each group. A notable example is the evolutionarily conserved neural-expressed miR-125b, which targets both VAChT and AChE-R (Table 2) and associates with all five groups: inflammation and anxiety (Manca et al., 2011; Xu et al., 2011; Danielsson et al., 2012; Matsukawa et al., 2013), brain damage (Rink and Khanna, 2011), neurodegenerative diseases (Lukiw and Alexandrov, 2012) and cardiac diseases (Voellenkle et al., 2010) and diverse pain syndromes (Imai et al., 2011; Kynast et al., 2013; Monastyrskaya et al., 2013; Sakai et al., 2013). Known association of each of these diseases with impaired cholinergic signaling (Shenhar-Tsarfaty et al., 2013a, b) supports the notion of physiological significance for surveillance by the overlapping miRNAs. A primate-specific example involves miR-765, which co-targets AChE-R and VAChT. Redell (Redell et al., 2010) found that miR-765 is upregulated in the plasma of patients after traumatic brain injury, compatible with changes in serum cholinesterases in post-stroke patients (Ben Assayag et al., 2010). Additionally, miR-765 targets the neurotrophin-3 receptor 3′UTR, and Muiños-Gimeno (Muinos-Gimeno et al., 2009) discovered a single nucleotide change located in the miR-765 binding site of this receptor's mRNA 3′UTR to be associated with panic disorder. Neurotrophin receptors regulate cholinergic signaling (Naumann et al., 2002), which associates with stress reactions (Kaufer et al., 1998; Sklan et al., 2004) as well as with RNA metabolism changes in the Alzheimer's brain (Berson et al., 2012; Lau et al., 2013); predicting that disrupted association of miRNAs with these receptors would impair cholinergic signaling and could increase the risk of anxiety-related diseases.

**Figure 3:**
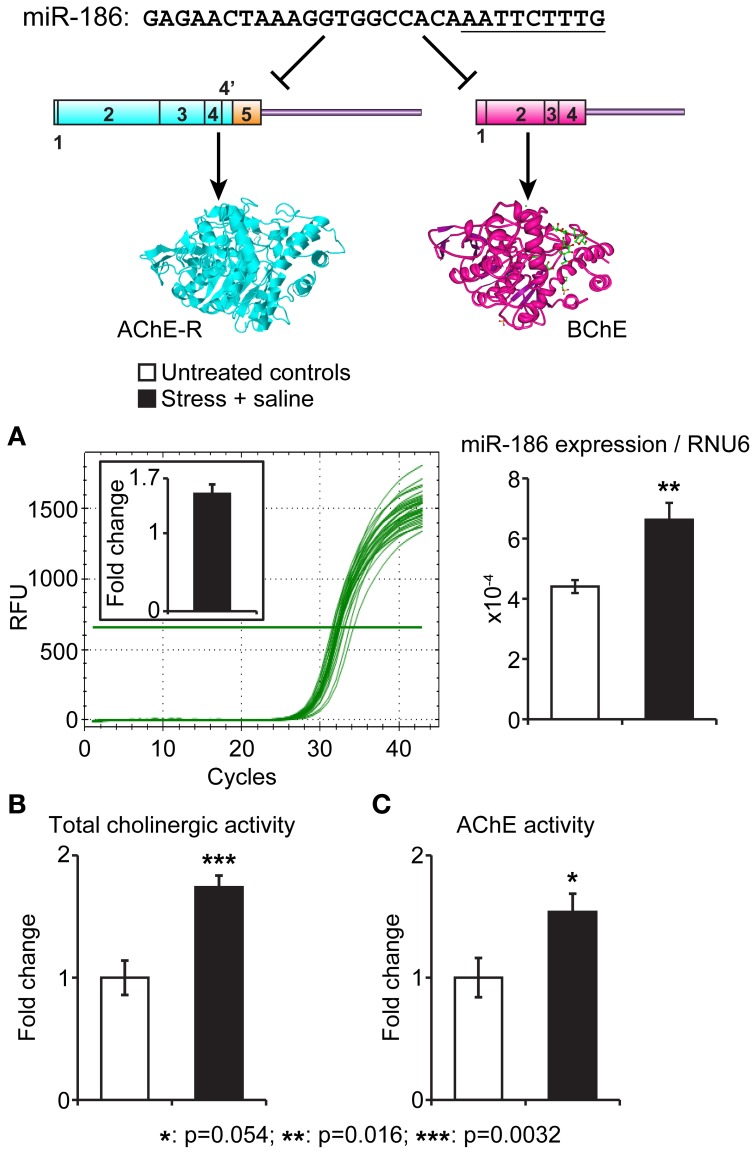
**Intestinal miR-186 increases under predator scent stress are accompanied by elevated BChE and AChE activities**. Top: The nucleotide sequence of the BChE and AChE-R-targeting miR-186 and schemes of its AChE-R and BChE mRNA targets. The “seed” sequence is underlined. **(A)** qRT-PCR quantification normalized to RNU6 levels demonstrates reproducible RFU values and 1.6-fold excess of miR-186 in intestinal sections from stressed mice, 7 days post-predator scent exposure (*p* = 0.016). **(B,C)** Highly significant intestinal elevation of total acetylthiocholine hydrolytic activity (*p* = 0.0032) accompanied by less pronounced AChE activity measured in the presence of 10 μM iso-OMPA (*p* = 0.05). *N* = 5 mice per group, in all tests.

To experimentally test for *in vivo* association of stress-induced changes in the identified miRNAs and their putative target genes, we selected the evolutionarily conserved miR-186 which predictably targets the two soluble cholinesterases, BChE and AChE-R. Of those, AChE-R increases under psychological stress were well documented (Kaufer et al., 1998; Cohen et al., 2002; Meshorer et al., 2005; Shaltiel et al., 2013), including the serum (Sklan et al., 2004), but BChE's involvement was never approached. In our current study, we quantified miR-186 levels in intestinal biopsies prepared from male C57BJ mice 7 days following 10 min exposure to cat litter (predator scent stress) and injection for four consecutive days with 50 μ g kg^−1^ saline (Zimmerman et al., 2012) compared to matched controls (*n* = 5 mice per group). In the intestinal biopsies, miR-186 expression normalized to the house-keeping short RNA RNU6 showed a 1.6-fold increase (*p* < 0.016) in pre-stressed mice. In parallel, these mice showed a 1.8-fold elevation in total cholinesterase activities (*p* < 0.003, Student's *t* test) as well as a less pronounced 1.6-fold increase in AChE levels (*p* < 0.054). Figure 3 presents these findings, demonstrating directly associated changes in the intestinal levels of the miR-186 and its two cholinergic regulating targets.

## Discussion

Combined use of four different bioinformatics algorithms identified a large number of miRNAs putatively targeting the 3′UTRs of ChAT, VAChT, AChE-S, AChE-R, and BChE. MiRNAs can notably regulate whole biological pathways; for example, miR-181 controls mouse hematopoiesis (Chen et al., 2004), miR-608 targets two inflammation-related transcripts, CDC42 and IL6 (Jeyapalan et al., 2011; Kang et al., 2011) and miR-221 controls multiple cancer pathways (Lupini et al., 2013). To challenge the possibility that certain miRNAs likewise regulate ACh metabolism and belong to the family of CholinomiRs, we searched for miRNAs targeting more than one of the five transcripts involved in the process of ACh synthesis, packaging and degradation. Intriguingly, packaging more than synthesis of ACh emerged as being co-regulated with its degradation, suggesting a dynamically controlled surveillance of these two processes. Furthermore, these findings highlight the option of differential post-transcriptional regulation of VAChT and ChAT, which share the same promoter but have distinct 3′-UTR domains.

The numbers of miRNAs we identified are likely to be under-estimated due to the exclusion of all the cancer-related miRNAs, which may be linked to cholinergic signaling as well. However, it is noteworthy that many of the miRNAs associated with ACh metabolism are primate-specific. This implies that there are no mouse models to study their function and influence, decreasing the likelihood of experimental animal studies of these miRNAs. Nevertheless, we found many human disease-association studies that demonstrate putative links of these miRNAs to inflammation and possibly reflecting the inflammatory blockade by ACh (Tracey, 2010). In comparison, only a few of the identified miRNAs appear to be largely modified in neurodegenerative diseases such as Alzheimer's disease, perhaps because cholinergic neurons decline early in the Alzheimer's brains (Mcgeer et al., 1984), so that it may be too late to find miRNAs playing a role in ACh metabolism in late stage brain samples from Alzheimer's patients. An exception is the AChE-targeted miR-132, which shows a drastic decline in the Alzheimer's brain (Lau et al., 2013).

The apparent relevance of CholinomiRs to diverse pain syndromes is particularly intriguing (Kress et al., 2013). Thus miR-199a-5p is expressed in the bladder's smooth muscle and urothelium and may play a role in bladder pain syndrome (Monastyrskaya et al., 2013) by suppressing LIN7C, ARHGAP12, PALS1, RND1 and PVRL1. In addition, miR-199a-5p is predicted to target both BChE and AChE-R, suggesting that its increase would up-regulate cholinergic stimulation in the bladder, which could also contribute to pain reactions. Likewise, miR-200b and miR-429 predictably target BChE and AChE-R, and changes in their levels were reported under neuropathic pain following sciatic nerve ligation within the limbic forebrain's nucleus accumbens (Imai et al., 2011). This has been attributed to miR-200b/429 targeting of DNA methyltransferase 3a (DNMT3a), which indeed accumulated in post-synaptic neurons in the nucleus accumbens under a neuropathic pain-like state. Such an increase may lead to long-term silencing of several genes at the transcriptional level, and enhanced cholinergic stimulation might contribute to this effect. An inverse effect has intriguingly been observed for miR-7a, which predictably targets VAChT and AChE-R and may hence reduce the packaging efficiency of ACh and **limit** cholinergic signals: miR-7a alleviates the maintenance of neuropathic pain through regulating neuronal excitability by targeting the β 2 subunit of the voltage-dependent sodium channel (Sakai et al., 2013). In all of these cases, the cholinergic targets may thus modulate the observed pain-related effects.

Our experimental test of miR-186 relevance for stress-related conditions revealed a direct dual association between elevated miR-186 and parallel increases in BChE levels in intestinal tissues from predator scent-injection-stressed mice. These effects were long-lasting and were only significant for BChE, highlighting for the first time, the changes in this protein as associated with psychological stress. That both miR-186 and its BChE target show intestinal increases under stress may indicate that these miRNA changes reflect a feedback response limiting excessive ACh stimulation; supporting this notion, serum BChE increases in post-stroke patients were associated with better prospects of recovery (Shenhar-Tsarfaty et al., 2013a). Further studies will be required to explore the molecular mechanisms underlying this involvement.

Several limitations need to be taken into account regarding this study. First, the search algorithms for miRNA candidates appear to differ substantially, each yielding different results. In our current study, we studied those miRNAs found in each of these algorithms, to improve the prospects of identifying as many relevant miRNAs as possible. Second, as our study spanned all of the miRNAs that predictably target the 3′-UTRs in all of the transcripts of interest, further studies will be required to functionally validate these miRNAs not only as single targeting but also as dually targeting more than one of these ACh metabolism-related transcripts. Third, we utilized a data-mining approach as before (Hanin and Soreq, 2011), and relied on explorative studies which link the identified miRNAs to disease association, but it remains unclear if such associations reflect the disease outcome or inversely, an effort of the system to protect itself from the disease.

Taken together, our current study highlights the importance of interrogating the extent and dynamics of miRNA regulation at a pathway level as a novel approach for studying the molecular mechanisms underlying specific processes in health and disease. Moreover, the large fraction of primate-specific miRNAs that were identified in our study calls for paying special attention to such cases. Given that miRNAs are considered “druggable” molecules, for example by antisense oligonucleotide manipulations (Shaked et al., 2009; Shaltiel et al., 2013), it is imperative to search for high throughput datasets from human tissue studies of relevant diseases and perhaps engineer new mouse models with knocked-in primate-specific miRNAs, such as miR-608. This can become a novel approach for identifying targets for therapeutic intervention with diseases where primate-specific miRNAs are subjected to major changes. In either case, our current approach represents the peak of an iceberg; however, it provides an initial proof of principle for the concept of joint regulation over different transcripts involved in specific neurotransmission pathways. This study should further be regarded as a first step in a long pathway, since we only focused on five transcripts out of many involved in only one pathway, the cholinergic signaling pathway; but there are many more transcripts, such as neurotrophin or nicotinic and muscarinic ACh receptors playing a role in cholinergic signaling. Our findings thus indicate overlapping miRNA regulation as a new surveillance mechanism that can balance cholinergic neurotransmission and may be of value for both basic and translational aspects of neuroinflammation-related disorders.

### Conflict of interest statement

The authors declare that the research was conducted in the absence of any commercial or financial relationships that could be construed as a potential conflict of interest.

## Footnotes

^1^http://www.ncbi.nlm.nih.gov/
