# Supplementary material for: Predicted overlapping microRNA regulators of acetylcholine packaging and degradation in neuroinflammation-related disorders
Source: Front Mol Neurosci. 2014 Feb 10;7:9. doi: 10.3389/fnmol.2014.00009 (PMC3918661; doi:10.3389/fnmol.2014.00009)
Supplement: Supplementary file 7 [file DataSheet7.DOCX]

**Supplementary Bibliography**

Abdellatif, M. (2011). Cardioprotective microRNAs. *Pediatr Cardiol* 32**,** 311-316.

Abrahams, Y., Laguette, M.J., Prince, S., and Collins, M. (2013). Polymorphisms within the COL5A1 3'-UTR that alters mRNA structure and the MIR608 gene are associated with Achilles tendinopathy. *Ann Hum Genet* 77**,** 204-214.

Ahmed, F.E., Ahmed, N.C., Vos, P.W., Bonnerup, C., Atkins, J.N., Casey, M., Nuovo, G.J., Naziri, W., Wiley, J.E., Mota, H., and Allison, R.R. (2013). Diagnostic microRNA markers to screen for sporadic human colon cancer in stool: I. Proof of principle. *Cancer Genomics Proteomics* 10**,** 93-113.

Akbas, F., Coskunpinar, E., Aynaci, E., Oltulu, Y.M., and Yildiz, P. (2012). Analysis of serum micro-RNAs as potential biomarker in chronic obstructive pulmonary disease. *Exp Lung Res* 38**,** 286-294.

Akcakaya, P., Ekelund, S., Kolosenko, I., Caramuta, S., Ozata, D.M., Xie, H., Lindforss, U., Olivecrona, H., and Lui, W.O. (2011). miR-185 and miR-133b deregulation is associated with overall survival and metastasis in colorectal cancer. *Int J Oncol* 39**,** 311-318.

Alexander, M.S., Kawahara, G., Motohashi, N., Casar, J.C., Eisenberg, I., Myers, J.A., Gasperini, M.J., Estrella, E.A., Kho, A.T., Mitsuhashi, S., Shapiro, F., Kang, P.B., and Kunkel, L.M. (2013). MicroRNA-199a is induced in dystrophic muscle and affects WNT signaling, cell proliferation, and myogenic differentiation. *Cell Death Differ* 20**,** 1194-1208.

Ali, S., Saleh, H., Sethi, S., Sarkar, F.H., and Philip, P.A. (2012). MicroRNA profiling of diagnostic needle aspirates from patients with pancreatic cancer. *Br J Cancer* 107**,** 1354-1360.

Alvarez-Mora, M.I., Rodriguez-Revenga, L., Madrigal, I., Torres-Silva, F., Mateu-Huertas, E., Lizano, E., Friedlander, M.R., Marti, E., Estivill, X., and Mila, M. (2013). MicroRNA expression profiling in blood from fragile X-associated tremor/ataxia syndrome patients. *Genes Brain Behav* 12**,** 595-603.

Andolfo, I., Liguori, L., De Antonellis, P., Cusanelli, E., Marinaro, F., Pistollato, F., Garzia, L., De Vita, G., Petrosino, G., Accordi, B., Migliorati, R., Basso, G., Iolascon, A., Cinalli, G., and Zollo, M. (2012). The micro-RNA 199b-5p regulatory circuit involves Hes1, CD15, and epigenetic modifications in medulloblastoma. *Neuro Oncol* 14**,** 596-612.

Archacki, S.R., Angheloiu, G., Tian, X.L., Tan, F.L., Dipaola, N., Shen, G.Q., Moravec, C., Ellis, S., Topol, E.J., and Wang, Q. (2003). Identification of new genes differentially expressed in coronary artery disease by expression profiling. *Physiol Genomics* 15**,** 65-74.

Armeanu-Ebinger, S., Herrmann, D., Bonin, M., Leuschner, I., Warmann, S.W., Fuchs, J., and Seitz, G. (2012). Differential expression of miRNAs in rhabdomyosarcoma and malignant rhabdoid tumor. *Exp Cell Res* 318**,** 2567-2577.

Bala, S., and Szabo, G. (2012). MicroRNA Signature in Alcoholic Liver Disease. *Int J Hepatol* 2012**,** 498232.

Balatti, V., Maniero, S., Ferracin, M., Veronese, A., Negrini, M., Ferrocci, G., Martini, F., and Tognon, M.G. (2011). MicroRNAs dysregulation in human malignant pleural mesothelioma. *J Thorac Oncol* 6**,** 844-851.

Bhattacharyya, M., and Bandyopadhyay, S. (2013). Studying the differential co-expression of microRNAs reveals significant role of white matter in early Alzheimer's progression. *Mol Biosyst* 9**,** 457-466.

Bimpaki, E.I., Iliopoulos, D., Moraitis, A., and Stratakis, C.A. (2010). MicroRNA signature in massive macronodular adrenocortical disease and implications for adrenocortical tumourigenesis. *Clin Endocrinol (Oxf)* 72**,** 744-751.

Boldrup, L., Coates, P.J., Wahlgren, M., Laurell, G., and Nylander, K. (2012). Subsite-based alterations in miR-21, miR-125b, and miR-203 in squamous cell carcinoma of the oral cavity and correlation to important target proteins. *J Carcinog* 11**,** 18.

Boren, T., Xiong, Y., Hakam, A., Wenham, R., Apte, S., Wei, Z., Kamath, S., Chen, D.T., Dressman, H., and Lancaster, J.M. (2008). MicroRNAs and their target messenger RNAs associated with endometrial carcinogenesis. *Gynecol Oncol* 110**,** 206-215.

Bostjancic, E., Zidar, N., and Glavac, D. (2009). MicroRNA microarray expression profiling in human myocardial infarction. *Dis Markers* 27**,** 255-268.

Bottoni, A., Zatelli, M.C., Ferracin, M., Tagliati, F., Piccin, D., Vignali, C., Calin, G.A., Negrini, M., Croce, C.M., and Degli Uberti, E.C. (2007). Identification of differentially expressed microRNAs by microarray: a possible role for microRNA genes in pituitary adenomas. *J Cell Physiol* 210**,** 370-377.

Bousquet, M., Harris, M.H., Zhou, B., and Lodish, H.F. (2010). MicroRNA miR-125b causes leukemia. *Proc Natl Acad Sci U S A* 107**,** 21558-21563.

Bozdag, S., Li, A., Riddick, G., Kotliarov, Y., Baysan, M., Iwamoto, F.M., Cam, M.C., Kotliarova, S., and Fine, H.A. (2013). Age-specific signatures of glioblastoma at the genomic, genetic, and epigenetic levels. *PLoS One* 8**,** e62982.

Cai, J., Wu, J., Zhang, H., Fang, L., Huang, Y., Yang, Y., Zhu, X., Li, R., and Li, M. (2013). miR-186 downregulation correlates with poor survival in lung adenocarcinoma, where it interferes with cell-cycle regulation. *Cancer Res* 73**,** 756-766.

Cao, P., Zhou, L., Zhang, J., Zheng, F., Wang, H., Ma, D., and Tian, J. (2013). Comprehensive expression profiling of microRNAs in laryngeal squamous cell carcinoma. *Head Neck* 35**,** 720-728.

Chan, Y.C., Roy, S., Huang, Y., Khanna, S., and Sen, C.K. (2012). The microRNA miR-199a-5p down-regulation switches on wound angiogenesis by derepressing the v-ets erythroblastosis virus E26 oncogene homolog 1-matrix metalloproteinase-1 pathway. *J Biol Chem* 287**,** 41032-41043.

Chen-Plotkin, A.S., Unger, T.L., Gallagher, M.D., Bill, E., Kwong, L.K., Volpicelli-Daley, L., Busch, J.I., Akle, S., Grossman, M., Van Deerlin, V., Trojanowski, J.Q., and Lee, V.M. (2012). TMEM106B, the risk gene for frontotemporal dementia, is regulated by the microRNA-132/212 cluster and affects progranulin pathways. *J Neurosci* 32**,** 11213-11227.

Chen, L., Li, Y., Fu, Y., Peng, J., Mo, M.H., Stamatakos, M., Teal, C.B., Brem, R.F., Stojadinovic, A., Grinkemeyer, M., Mccaffrey, T.A., Man, Y.G., and Fu, S.W. (2013a). Role of deregulated microRNAs in breast cancer progression using FFPE tissue. *PLoS One* 8**,** e54213.

Chen, L., Zhang, A., Li, Y., Zhang, K., Han, L., Du, W., Yan, W., Li, R., Wang, Y., Wang, K., Pu, P., Jiang, T., Jiang, C., and Kang, C. (2013b). MiR-24 regulates the proliferation and invasion of glioma by ST7L via beta-catenin/Tcf-4 signaling. *Cancer Lett* 329**,** 174-180.

Chen, N.X., Kiattisunthorn, K., O'neill, K.D., Chen, X., Moorthi, R.N., Gattone, V.H., 2nd, Allen, M.R., and Moe, S.M. (2013c). Decreased microRNA is involved in the vascular remodeling abnormalities in chronic kidney disease (CKD). *PLoS One* 8**,** e64558.

Chen, S., He, Y., Ding, J., Jiang, Y., Jia, S., Xia, W., Zhao, J., Lu, M., Gu, Z., and Gao, Y. (2010). An insertion/deletion polymorphism in the 3' untranslated region of beta-transducin repeat-containing protein (betaTrCP) is associated with susceptibility for hepatocellular carcinoma in Chinese. *Biochem Biophys Res Commun* 391**,** 552-556.

Chen, Y., Ge, W., Xu, L., Qu, C., Zhu, M., Zhang, W., and Xiao, Y. (2012). miR-200b is involved in intestinal fibrosis of Crohn's disease. *Int J Mol Med* 29**,** 601-606.

Cheung, H.H., Davis, A.J., Lee, T.L., Pang, A.L., Nagrani, S., Rennert, O.M., and Chan, W.Y. (2011). Methylation of an intronic region regulates miR-199a in testicular tumor malignancy. *Oncogene* 30**,** 3404-3415.

Chiang, K., Liu, H., and Rice, A.P. (2013). miR-132 enhances HIV-1 replication. *Virology* 438**,** 1-4.

Chiang, Y., Song, Y., Wang, Z., Liu, Z., Gao, P., Liang, J., Zhu, J., Xing, C., and Xu, H. (2012). microRNA-192, -194 and -215 are frequently downregulated in colorectal cancer. *Exp Ther Med* 3**,** 560-566.

Chung, Y.W., Bae, H.S., Song, J.Y., Lee, J.K., Lee, N.W., Kim, T., and Lee, K.W. (2013). Detection of microRNA as novel biomarkers of epithelial ovarian cancer from the serum of ovarian cancer patient. *Int J Gynecol Cancer* 23**,** 673-679.

Coppola, N., Potenza, N., Pisaturo, M., Mosca, N., Tonziello, G., Signoriello, G., Messina, V., Sagnelli, C., Russo, A., and Sagnelli, E. (2013). Liver microRNA hsa-miR-125a-5p in HBV chronic infection: correlation with HBV replication and disease progression. *PLoS One* 8**,** e65336.

Costa, F.F., Bischof, J.M., Vanin, E.F., Lulla, R.R., Wang, M., Sredni, S.T., Rajaram, V., Bonaldo Mde, F., Wang, D., Goldman, S., Tomita, T., and Soares, M.B. (2011). Identification of microRNAs as potential prognostic markers in ependymoma. *PLoS One* 6**,** e25114.

Cui, E.H., Li, H.J., Hua, F., Wang, B., Mao, W., Feng, X.R., Li, J.Y., and Wang, X. (2013). Serum microRNA 125b as a diagnostic or prognostic biomarker for advanced NSCLC patients receiving cisplatin-based chemotherapy. *Acta Pharmacol Sin* 34**,** 309-313.

Dai, Y., Sui, W., Lan, H., Yan, Q., Huang, H., and Huang, Y. (2009). Comprehensive analysis of microRNA expression patterns in renal biopsies of lupus nephritis patients. *Rheumatol Int* 29**,** 749-754.

Dai, Y., Xia, W., Song, T., Su, X., Li, J., Li, S., Chen, Y., Wang, W., Ding, H., Liu, X., Li, H., Zhao, Q., and Shao, N. (2013). MicroRNA-200b is overexpressed in endometrial adenocarcinomas and enhances MMP2 activity by downregulating TIMP2 in human endometrial cancer cell line HEC-1A cells. *Nucleic Acid Ther* 23**,** 29-34.

Dallaire, A., Garand, C., Paquel, E.R., Mitchell, S.J., De Cabo, R., Simard, M.J., and Lebel, M. (2012). Down regulation of miR-124 in both Werner syndrome DNA helicase mutant mice and mutant Caenorhabditis elegans wrn-1 reveals the importance of this microRNA in accelerated aging. *Aging (Albany NY)* 4**,** 636-647.

Danielsson, K., Wahlin, Y.B., Gu, X., Boldrup, L., and Nylander, K. (2012). Altered expression of miR-21, miR-125b, and miR-203 indicates a role for these microRNAs in oral lichen planus. *J Oral Pathol Med* 41**,** 90-95.

De Felice, B., Guida, M., Guida, M., Coppola, C., De Mieri, G., and Cotrufo, R. (2012). A miRNA signature in leukocytes from sporadic amyotrophic lateral sclerosis. *Gene* 508**,** 35-40.

Deng, Y., Zhao, J., Sakurai, D., Kaufman, K.M., Edberg, J.C., Kimberly, R.P., Kamen, D.L., Gilkeson, G.S., Jacob, C.O., Scofield, R.H., Langefeld, C.D., Kelly, J.A., Ramsey-Goldman, R., Petri, M.A., Reveille, J.D., Vila, L.M., Alarcon, G.S., Vyse, T.J., Pons-Estel, B.A., Argentine Collaborative, G., Freedman, B.I., Gaffney, P.M., Sivils, K.M., James, J.A., Gregersen, P.K., Anaya, J.M., Niewold, T.B., Merrill, J.T., Criswell, L.A., Stevens, A.M., Boackle, S.A., Cantor, R.M., Chen, W., Grossman, J.M., Hahn, B.H., Harley, J.B., Alarcomicronn-Riquelme, M.E., Biolupus, Networks, G., Brown, E.E., and Tsao, B.P. (2013). MicroRNA-3148 modulates allelic expression of toll-like receptor 7 variant associated with systemic lupus erythematosus. *PLoS Genet* 9**,** e1003336.

Diaz-Prado, S., Cicione, C., Muinos-Lopez, E., Hermida-Gomez, T., Oreiro, N., Fernandez-Lopez, C., and Blanco, F.J. (2012). Characterization of microRNA expression profiles in normal and osteoarthritic human chondrocytes. *BMC Musculoskelet Disord* 13**,** 144.

Dickinson, B.A., Semus, H.M., Montgomery, R.L., Stack, C., Latimer, P.A., Lewton, S.M., Lynch, J.M., Hullinger, T.G., Seto, A.G., and Van Rooij, E. (2013). Plasma microRNAs serve as biomarkers of therapeutic efficacy and disease progression in hypertension-induced heart failure. *Eur J Heart Fail* 15**,** 650-659.

Doeppner, T.R., Doehring, M., Bretschneider, E., Zechariah, A., Kaltwasser, B., Muller, B., Koch, J.C., Bahr, M., Hermann, D.M., and Michel, U. (2013). MicroRNA-124 protects against focal cerebral ischemia via mechanisms involving Usp14-dependent REST degradation. *Acta Neuropathol* 126**,** 251-265.

Duan, Q., Wang, X., Gong, W., Ni, L., Chen, C., He, X., Chen, F., Yang, L., Wang, P., and Wang, D.W. (2012). ER stress negatively modulates the expression of the miR-199a/214 cluster to regulates tumor survival and progression in human hepatocellular cancer. *PLoS One* 7**,** e31518.

Duncavage, E., Goodgame, B., Sezhiyan, A., Govindan, R., and Pfeifer, J. (2010). Use of microRNA expression levels to predict outcomes in resected stage I non-small cell lung cancer. *J Thorac Oncol* 5**,** 1755-1763.

Dutta, R., Chomyk, A.M., Chang, A., Ribaudo, M.V., Deckard, S.A., Doud, M.K., Edberg, D.D., Bai, B., Li, M., Baranzini, S.E., Fox, R.J., Staugaitis, S.M., Macklin, W.B., and Trapp, B.D. (2013). Hippocampal demyelination and memory dysfunction are associated with increased levels of the neuronal microRNA miR-124 and reduced AMPA receptors. *Ann Neurol* 73**,** 637-645.

El Tayebi, H.M., Hosny, K.A., Esmat, G., Breuhahn, K., and Abdelaziz, A.I. (2012). miR-615-5p is restrictedly expressed in cirrhotic and cancerous liver tissues and its overexpression alleviates the tumorigenic effects in hepatocellular carcinoma. *FEBS Lett* 586**,** 3309-3316.

Erkan, E.P., Breakefield, X.O., and Saydam, O. (2011). miRNA signature of schwannomas: possible role(s) of "tumor suppressor" miRNAs in benign tumors. *Oncotarget* 2**,** 265-270.

Eskildsen, T.V., Jeppesen, P.L., Schneider, M., Nossent, A.Y., Sandberg, M.B., Hansen, P.B., Jensen, C.H., Hansen, M.L., Marcussen, N., Rasmussen, L.M., Bie, P., Andersen, D.C., and Sheikh, S.P. (2013). Angiotensin II Regulates microRNA-132/-212 in Hypertensive Rats and Humans. *Int J Mol Sci* 14**,** 11190-11207.

Estep, M., Armistead, D., Hossain, N., Elarainy, H., Goodman, Z., Baranova, A., Chandhoke, V., and Younossi, Z.M. (2010). Differential expression of miRNAs in the visceral adipose tissue of patients with non-alcoholic fatty liver disease. *Aliment Pharmacol Ther* 32**,** 487-497.

Etoh, M., Jinnin, M., Makino, K., Yamane, K., Nakayama, W., Aoi, J., Honda, N., Kajihara, I., Makino, T., Fukushima, S., and Ihn, H. (2013). microRNA-7 down-regulation mediates excessive collagen expression in localized scleroderma. *Arch Dermatol Res* 305**,** 9-15.

Fang, Y., Xue, J.L., Shen, Q., Chen, J., and Tian, L. (2012). MicroRNA-7 inhibits tumor growth and metastasis by targeting the phosphoinositide 3-kinase/Akt pathway in hepatocellular carcinoma. *Hepatology* 55**,** 1852-1862.

Farazi, T.A., Horlings, H.M., Ten Hoeve, J.J., Mihailovic, A., Halfwerk, H., Morozov, P., Brown, M., Hafner, M., Reyal, F., Van Kouwenhove, M., Kreike, B., Sie, D., Hovestadt, V., Wessels, L.F., Van De Vijver, M.J., and Tuschl, T. (2011). MicroRNA sequence and expression analysis in breast tumors by deep sequencing. *Cancer Res* 71**,** 4443-4453.

Fassan, M., Baffa, R., Palazzo, J.P., Lloyd, J., Crosariol, M., Liu, C.G., Volinia, S., Alder, H., Rugge, M., Croce, C.M., and Rosenberg, A. (2009). MicroRNA expression profiling of male breast cancer. *Breast Cancer Res* 11**,** R58.

Fassan, M., Pizzi, M., Realdon, S., Balistreri, M., Guzzardo, V., Zagonel, V., Castoro, C., Mastracci, L., Farinati, F., Nitti, D., Zaninotto, G., and Rugge, M. (2013). The HER2-miR125a5p/miR125b loop in gastric and esophageal carcinogenesis. *Hum Pathol* 44**,** 1804-1810.

Fassan, M., Volinia, S., Palatini, J., Pizzi, M., Baffa, R., De Bernard, M., Battaglia, G., Parente, P., Croce, C.M., Zaninotto, G., Ancona, E., and Rugge, M. (2011). MicroRNA expression profiling in human Barrett's carcinogenesis. *Int J Cancer* 129**,** 1661-1670.

Fassina, A., Cappellesso, R., and Fassan, M. (2011). Classification of non-small cell lung carcinoma in transthoracic needle specimens using microRNA expression profiling. *Chest* 140**,** 1305-1311.

Favreau, A.J., Cross, E.L., and Sathyanarayana, P. (2012). miR-199b-5p directly targets PODXL and DDR1 and decreased levels of miR-199b-5p correlate with elevated expressions of PODXL and DDR1 in acute myeloid leukemia. *Am J Hematol* 87**,** 442-446.

Ferrer, G., Navarro, A., Hodgson, K., Aymerich, M., Pereira, A., Baumann, T., Monzo, M., Moreno, C., and Montserrat, E. (2013). MicroRNA expression in chronic lymphocytic leukemia developing autoimmune hemolytic anemia. *Leuk Lymphoma* 54**,** 2016-2022.

Foekens, J.A., Sieuwerts, A.M., Smid, M., Look, M.P., De Weerd, V., Boersma, A.W., Klijn, J.G., Wiemer, E.A., and Martens, J.W. (2008). Four miRNAs associated with aggressiveness of lymph node-negative, estrogen receptor-positive human breast cancer. *Proc Natl Acad Sci U S A* 105**,** 13021-13026.

Formosa, A., Lena, A.M., Markert, E.K., Cortelli, S., Miano, R., Mauriello, A., Croce, N., Vandesompele, J., Mestdagh, P., Finazzi-Agro, E., Levine, A.J., Melino, G., Bernardini, S., and Candi, E. (2013). DNA methylation silences miR-132 in prostate cancer. *Oncogene* 32**,** 127-134.

Fowler, A., Thomson, D., Giles, K., Maleki, S., Mreich, E., Wheeler, H., Leedman, P., Biggs, M., Cook, R., Little, N., Robinson, B., and Mcdonald, K. (2011). miR-124a is frequently down-regulated in glioblastoma and is involved in migration and invasion. *Eur J Cancer* 47**,** 953-963.

Fu, H.L., Wu De, P., Wang, X.F., Wang, J.G., Jiao, F., Song, L.L., Xie, H., Wen, X.Y., Shan, H.S., Du, Y.X., and Zhao, Y.P. (2013). Altered miRNA expression is associated with differentiation, invasion, and metastasis of esophageal squamous cell carcinoma (ESCC) in patients from Huaian, China. *Cell Biochem Biophys* 67**,** 657-668.

Furuta, M., Kozaki, K.I., Tanaka, S., Arii, S., Imoto, I., and Inazawa, J. (2010). miR-124 and miR-203 are epigenetically silenced tumor-suppressive microRNAs in hepatocellular carcinoma. *Carcinogenesis* 31**,** 766-776.

Gandhi, R., Healy, B., Gholipour, T., Egorova, S., Musallam, A., Hussain, M.S., Nejad, P., Patel, B., Hei, H., Khoury, S., Quintana, F., Kivisakk, P., Chitnis, T., and Weiner, H.L. (2013). Circulating microRNAs as biomarkers for disease staging in multiple sclerosis. *Ann Neurol* 73**,** 729-740.

Gao, W., Shen, H., Liu, L., Xu, J., Xu, J., and Shu, Y. (2011). MiR-21 overexpression in human primary squamous cell lung carcinoma is associated with poor patient prognosis. *J Cancer Res Clin Oncol* 137**,** 557-566.

Gao, W., Yu, Y., Cao, H., Shen, H., Li, X., Pan, S., and Shu, Y. (2010). Deregulated expression of miR-21, miR-143 and miR-181a in non small cell lung cancer is related to clinicopathologic characteristics or patient prognosis. *Biomed Pharmacother* 64**,** 399-408.

Gatsiou, A., Boeckel, J.N., Randriamboavonjy, V., and Stellos, K. (2012). MicroRNAs in platelet biogenesis and function: implications in vascular homeostasis and inflammation. *Curr Vasc Pharmacol* 10**,** 524-531.

Gebauer, K., Peters, I., Dubrowinskaja, N., Hennenlotter, J., Abbas, M., Scherer, R., Tezval, H., Merseburger, A.S., Stenzl, A., Kuczyk, M.A., and Serth, J. (2013). Hsa-mir-124-3 CpG island methylation is associated with advanced tumours and disease recurrence of patients with clear cell renal cell carcinoma. *Br J Cancer* 108**,** 131-138.

Giangreco, A.A., Vaishnav, A., Wagner, D., Finelli, A., Fleshner, N., Van Der Kwast, T., Vieth, R., and Nonn, L. (2013). Tumor suppressor microRNAs, miR-100 and -125b, are regulated by 1,25-dihydroxyvitamin D in primary prostate cells and in patient tissue. *Cancer Prev Res (Phila)* 6**,** 483-494.

Giles, K.M., Brown, R.A., Epis, M.R., Kalinowski, F.C., and Leedman, P.J. (2013). miRNA-7-5p inhibits melanoma cell migration and invasion. *Biochem Biophys Res Commun* 430**,** 706-710.

Godwin, J.G., Ge, X., Stephan, K., Jurisch, A., Tullius, S.G., and Iacomini, J. (2010). Identification of a microRNA signature of renal ischemia reperfusion injury. *Proc Natl Acad Sci U S A* 107**,** 14339-14344.

Goeppert, B., Schmezer, P., Dutruel, C., Oakes, C., Renner, M., Breinig, M., Warth, A., Vogel, M.N., Mittelbronn, M., Mehrabi, A., Gdynia, G., Penzel, R., Longerich, T., Breuhahn, K., Popanda, O., Plass, C., Schirmacher, P., and Kern, M.A. (2010). Down-regulation of tumor suppressor A kinase anchor protein 12 in human hepatocarcinogenesis by epigenetic mechanisms. *Hepatology* 52**,** 2023-2033.

Goren, Y., Kushnir, M., Zafrir, B., Tabak, S., Lewis, B.S., and Amir, O. (2012). Serum levels of microRNAs in patients with heart failure. *Eur J Heart Fail* 14**,** 147-154.

Gottardo, F., Liu, C.G., Ferracin, M., Calin, G.A., Fassan, M., Bassi, P., Sevignani, C., Byrne, D., Negrini, M., Pagano, F., Gomella, L.G., Croce, C.M., and Baffa, R. (2007). Micro-RNA profiling in kidney and bladder cancers. *Urol Oncol* 25**,** 387-392.

Gougelet, A., and Colnot, S. (2013). MicroRNA-feedback loop as a key modulator of liver tumorigenesis and inflammation. *World J Gastroenterol* 19**,** 440-444.

Guerau-De-Arellano, M., Alder, H., Ozer, H.G., Lovett-Racke, A., and Racke, M.K. (2012). miRNA profiling for biomarker discovery in multiple sclerosis: from microarray to deep sequencing. *J Neuroimmunol* 248**,** 32-39.

Guglielmelli, P., Tozzi, L., Pancrazzi, A., Bogani, C., Antonioli, E., Ponziani, V., Poli, G., Zini, R., Ferrari, S., Manfredini, R., Bosi, A., Vannucchi, A.M., and Consortium, M.P.D.R. (2007). MicroRNA expression profile in granulocytes from primary myelofibrosis patients. *Exp Hematol* 35**,** 1708-1718.

Guled, M., Lahti, L., Lindholm, P.M., Salmenkivi, K., Bagwan, I., Nicholson, A.G., and Knuutila, S. (2009). CDKN2A, NF2, and JUN are dysregulated among other genes by miRNAs in malignant mesothelioma -A miRNA microarray analysis. *Genes Chromosomes Cancer* 48**,** 615-623.

Guo, J., Dong, Q., Fang, Z., Chen, X., Lu, H., Wang, K., Yin, Y., Cai, X., Zhao, N., Chen, J., Zen, K., Zhang, J., and Zhang, C.Y. (2010). Identification of miRNAs that are associated with tumor metastasis in neuroblastoma. *Cancer Biol Ther* 9**,** 446-452.

Guo, J., Miao, Y., Xiao, B., Huan, R., Jiang, Z., Meng, D., and Wang, Y. (2009). Differential expression of microRNA species in human gastric cancer versus non-tumorous tissues. *J Gastroenterol Hepatol* 24**,** 652-657.

Guo, Y., Fu, W., Chen, H., Shang, C., and Zhong, M. (2012). miR-24 functions as a tumor suppressor in Hep2 laryngeal carcinoma cells partly through down-regulation of the S100A8 protein. *Oncol Rep* 27**,** 1097-1103.

Gupta, M.K., Halley, C., Duan, Z.H., Lappe, J., Viterna, J., Jana, S., Augoff, K., Mohan, M.L., Vasudevan, N.T., Na, J., Sossey-Alaoui, K., Liu, X., Liu, C.G., Tang, W.H., and Naga Prasad, S.V. (2013). miRNA-548c: a specific signature in circulating PBMCs from dilated cardiomyopathy patients. *J Mol Cell Cardiol* 62**,** 131-141.

Hamfjord, J., Stangeland, A.M., Hughes, T., Skrede, M.L., Tveit, K.M., Ikdahl, T., and Kure, E.H. (2012). Differential expression of miRNAs in colorectal cancer: comparison of paired tumor tissue and adjacent normal mucosa using high-throughput sequencing. *PLoS One* 7**,** e34150.

Han, B., Shi, X., Peng, Q., and Gao, W. (2012a). Study on Genetic Variance of miR-541 in Type 1 Diabetes. *ISRN Endocrinol* 2012**,** 630861.

Han, H.S., Yun, J., Lim, S.N., Han, J.H., Lee, K.H., Kim, S.T., Kang, M.H., Son, S.M., Lee, Y.M., Choi, S.Y., Yun, S.J., Kim, W.J., and Lee, O.J. (2013a). Downregulation of cell-free miR-198 as a diagnostic biomarker for lung adenocarcinoma-associated malignant pleural effusion. *Int J Cancer* 133**,** 645-652.

Han, Y., Chen, J., Zhao, X., Liang, C., Wang, Y., Sun, L., Jiang, Z., Zhang, Z., Yang, R., Chen, J., Li, Z., Tang, A., Li, X., Ye, J., Guan, Z., Gui, Y., and Cai, Z. (2011). MicroRNA expression signatures of bladder cancer revealed by deep sequencing. *PLoS One* 6**,** e18286.

Han, Z.B., Yang, Z., Chi, Y., Zhang, L., Wang, Y., Ji, Y., Wang, J., Zhao, H., and Han, Z.C. (2013b). MicroRNA-124 suppresses breast cancer cell growth and motility by targeting CD151. *Cell Physiol Biochem* 31**,** 823-832.

Han, Z.B., Zhong, L., Teng, M.J., Fan, J.W., Tang, H.M., Wu, J.Y., Chen, H.Y., Wang, Z.W., Qiu, G.Q., and Peng, Z.H. (2012b). Identification of recurrence-related microRNAs in hepatocellular carcinoma following liver transplantation. *Mol Oncol* 6**,** 445-457.

Hanieh, H., and Alzahrani, A. (2013). MicroRNA-132 suppresses autoimmune encephalomyelitis by inducing cholinergic anti-inflammation: a new Ahr-based exploration. *Eur J Immunol* 43**,** 2771-2782.

Hernandez, J.M., Elahi, A., Clark, C.W., Wang, J., Humphries, L.A., Centeno, B., Bloom, G., Fuchs, B.C., Yeatman, T., and Shibata, D. (2013). miR-675 Mediates Downregulation of Twist1 and Rb in AFP-Secreting Hepatocellular Carcinoma. *Ann Surg Oncol* 20 Suppl 3**,** 625-635.

Hoekstra, M., Van Der Lans, C.A., Halvorsen, B., Gullestad, L., Kuiper, J., Aukrust, P., Van Berkel, T.J., and Biessen, E.A. (2010). The peripheral blood mononuclear cell microRNA signature of coronary artery disease. *Biochem Biophys Res Commun* 394**,** 792-797.

Hong, M.J., Choi, Y.Y., Jang, J.A., Jung, H.J., Lee, S.Y., Lee, W.K., Yoo, S.S., Lee, J., Cha, S.I., Kim, C.H., Lee, E., Jeon, H.S., Son, J.W., and Park, J.Y. (2013). Association between genetic variants in pre-microRNAs and survival of early-stage NSCLC. *J Thorac Oncol* 8**,** 703-710.

Hu, Z.Y., Luo, J.F., Zhong, S.L., Xue, L., Chen, Y.F., and Fan, R.X. (2012). [MicroRNAs expression in normal and dissected aortic tissue]. *Zhonghua Xin Xue Guan Bing Za Zhi* 40**,** 406-410.

Huang, A.J., Yu, K.D., Li, J., Fan, L., and Shao, Z.M. (2012a). Polymorphism rs4919510:C>G in mature sequence of human microRNA-608 contributes to the risk of HER2-positive breast cancer but not other subtypes. *PLoS One* 7**,** e35252.

Huang, J.J., Yu, J., Li, J.Y., Liu, Y.T., and Zhong, R.Q. (2012b). Circulating microRNA expression is associated with genetic subtype and survival of multiple myeloma. *Med Oncol* 29**,** 2402-2408.

Huang, L., Lin, J.X., Yu, Y.H., Zhang, M.Y., Wang, H.Y., and Zheng, M. (2012c). Downregulation of six microRNAs is associated with advanced stage, lymph node metastasis and poor prognosis in small cell carcinoma of the cervix. *PLoS One* 7**,** e33762.

Huang, Y., Dai, Y., Yang, J., Chen, T., Yin, Y., Tang, M., Hu, C., and Zhang, L. (2009). Microarray analysis of microRNA expression in renal clear cell carcinoma. *Eur J Surg Oncol* 35**,** 1119-1123.

Huang, Y.S., Dai, Y., Yu, X.F., Bao, S.Y., Yin, Y.B., Tang, M., and Hu, C.X. (2008). Microarray analysis of microRNA expression in hepatocellular carcinoma and non-tumorous tissues without viral hepatitis. *J Gastroenterol Hepatol* 23**,** 87-94.

Hui, A.B., Lenarduzzi, M., Krushel, T., Waldron, L., Pintilie, M., Shi, W., Perez-Ordonez, B., Jurisica, I., O'sullivan, B., Waldron, J., Gullane, P., Cummings, B., and Liu, F.F. (2010). Comprehensive MicroRNA profiling for head and neck squamous cell carcinomas. *Clin Cancer Res* 16**,** 1129-1139.

Hur, K., Toiyama, Y., Takahashi, M., Balaguer, F., Nagasaka, T., Koike, J., Hemmi, H., Koi, M., Boland, C.R., and Goel, A. (2013). MicroRNA-200c modulates epithelial-to-mesenchymal transition (EMT) in human colorectal cancer metastasis. *Gut* 62**,** 1315-1326.

Iborra, M., Bernuzzi, F., Correale, C., Vetrano, S., Fiorino, G., Beltran, B., Marabita, F., Locati, M., Spinelli, A., Nos, P., Invernizzi, P., and Danese, S. (2013). Identification of serum and tissue micro-RNA expression profiles in different stages of inflammatory bowel disease. *Clin Exp Immunol* 173**,** 250-258.

Ichimi, T., Enokida, H., Okuno, Y., Kunimoto, R., Chiyomaru, T., Kawamoto, K., Kawahara, K., Toki, K., Kawakami, K., Nishiyama, K., Tsujimoto, G., Nakagawa, M., and Seki, N. (2009). Identification of novel microRNA targets based on microRNA signatures in bladder cancer. *Int J Cancer* 125**,** 345-352.

Imig, J., Motsch, N., Zhu, J.Y., Barth, S., Okoniewski, M., Reineke, T., Tinguely, M., Faggioni, A., Trivedi, P., Meister, G., Renner, C., and Grasser, F.A. (2011). microRNA profiling in Epstein-Barr virus-associated B-cell lymphoma. *Nucleic Acids Res* 39**,** 1880-1893.

Iorio, M.V., Visone, R., Di Leva, G., Donati, V., Petrocca, F., Casalini, P., Taccioli, C., Volinia, S., Liu, C.G., Alder, H., Calin, G.A., Menard, S., and Croce, C.M. (2007). MicroRNA signatures in human ovarian cancer. *Cancer Res* 67**,** 8699-8707.

Izzotti, A., Calin, G.A., Arrigo, P., Steele, V.E., Croce, C.M., and De Flora, S. (2009). Downregulation of microRNA expression in the lungs of rats exposed to cigarette smoke. *FASEB J* 23**,** 806-812.

Jia, L., Wu, J., Zhang, L., Chen, J., Zhong, D., Xu, S., Xie, C., and Cai, J. (2013). Restoration of miR-1228* expression suppresses epithelial-mesenchymal transition in gastric cancer. *PLoS One* 8**,** e58637.

Jiang, L., Huang, Q., Zhang, S., Zhang, Q., Chang, J., Qiu, X., and Wang, E. (2010). Hsa-miR-125a-3p and hsa-miR-125a-5p are downregulated in non-small cell lung cancer and have inverse effects on invasion and migration of lung cancer cells. *BMC Cancer* 10**,** 318.

Jin, J., Cheng, Y., Zhang, Y., Wood, W., Peng, Q., Hutchison, E., Mattson, M.P., Becker, K.G., and Duan, W. (2012a). Interrogation of brain miRNA and mRNA expression profiles reveals a molecular regulatory network that is perturbed by mutant huntingtin. *J Neurochem* 123**,** 477-490.

Jin, L., Hu, W.L., Jiang, C.C., Wang, J.X., Han, C.C., Chu, P., Zhang, L.J., Thorne, R.F., Wilmott, J., Scolyer, R.A., Hersey, P., Zhang, X.D., and Wu, M. (2011). MicroRNA-149*, a p53-responsive microRNA, functions as an oncogenic regulator in human melanoma. *Proc Natl Acad Sci U S A* 108**,** 15840-15845.

Jin, W., Reddy, M.A., Chen, Z., Putta, S., Lanting, L., Kato, M., Park, J.T., Chandra, M., Wang, C., Tangirala, R.K., and Natarajan, R. (2012b). Small RNA sequencing reveals microRNAs that modulate angiotensin II effects in vascular smooth muscle cells. *J Biol Chem* 287**,** 15672-15683.

Jones, S.W., Watkins, G., Le Good, N., Roberts, S., Murphy, C.L., Brockbank, S.M., Needham, M.R., Read, S.J., and Newham, P. (2009). The identification of differentially expressed microRNA in osteoarthritic tissue that modulate the production of TNF-alpha and MMP13. *Osteoarthritis Cartilage* 17**,** 464-472.

Jung, M., Mollenkopf, H.J., Grimm, C., Wagner, I., Albrecht, M., Waller, T., Pilarsky, C., Johannsen, M., Stephan, C., Lehrach, H., Nietfeld, W., Rudel, T., Jung, K., and Kristiansen, G. (2009). MicroRNA profiling of clear cell renal cell cancer identifies a robust signature to define renal malignancy. *J Cell Mol Med* 13**,** 3918-3928.

Junn, E., Lee, K.W., Jeong, B.S., Chan, T.W., Im, J.Y., and Mouradian, M.M. (2009). Repression of alpha-synuclein expression and toxicity by microRNA-7. *Proc Natl Acad Sci U S A* 106**,** 13052-13057.

Kahlert, C., Klupp, F., Brand, K., Lasitschka, F., Diederichs, S., Kirchberg, J., Rahbari, N., Dutta, S., Bork, U., Fritzmann, J., Reissfelder, C., Koch, M., and Weitz, J. (2011). Invasion front-specific expression and prognostic significance of microRNA in colorectal liver metastases. *Cancer Sci* 102**,** 1799-1807.

Kan, C.W., Hahn, M.A., Gard, G.B., Maidens, J., Huh, J.Y., Marsh, D.J., and Howell, V.M. (2012). Elevated levels of circulating microRNA-200 family members correlate with serous epithelial ovarian cancer. *BMC Cancer* 12**,** 627.

Karakatsanis, A., Papaconstantinou, I., Gazouli, M., Lyberopoulou, A., Polymeneas, G., and Voros, D. (2013). Expression of microRNAs, miR-21, miR-31, miR-122, miR-145, miR-146a, miR-200c, miR-221, miR-222, and miR-223 in patients with hepatocellular carcinoma or intrahepatic cholangiocarcinoma and its prognostic significance. *Mol Carcinog* 52**,** 297-303.

Katare, R., Riu, F., Mitchell, K., Gubernator, M., Campagnolo, P., Cui, Y., Fortunato, O., Avolio, E., Cesselli, D., Beltrami, A.P., Angelini, G., Emanueli, C., and Madeddu, P. (2011). Transplantation of human pericyte progenitor cells improves the repair of infarcted heart through activation of an angiogenic program involving micro-RNA-132. *Circ Res* 109**,** 894-906.

Ke, Y., Zhao, W., Xiong, J., and Cao, R. (2013). miR-149 Inhibits Non-Small-Cell Lung Cancer Cells EMT by Targeting FOXM1. *Biochem Res Int* 2013**,** 506731.

Keller, A., Backes, C., Leidinger, P., Kefer, N., Boisguerin, V., Barbacioru, C., Vogel, B., Matzas, M., Huwer, H., Katus, H.A., Stahler, C., Meder, B., and Meese, E. (2011a). Next-generation sequencing identifies novel microRNAs in peripheral blood of lung cancer patients. *Mol Biosyst* 7**,** 3187-3199.

Keller, A., Leidinger, P., Bauer, A., Elsharawy, A., Haas, J., Backes, C., Wendschlag, A., Giese, N., Tjaden, C., Ott, K., Werner, J., Hackert, T., Ruprecht, K., Huwer, H., Huebers, J., Jacobs, G., Rosenstiel, P., Dommisch, H., Schaefer, A., Muller-Quernheim, J., Wullich, B., Keck, B., Graf, N., Reichrath, J., Vogel, B., Nebel, A., Jager, S.U., Staehler, P., Amarantos, I., Boisguerin, V., Staehler, C., Beier, M., Scheffler, M., Buchler, M.W., Wischhusen, J., Haeusler, S.F., Dietl, J., Hofmann, S., Lenhof, H.P., Schreiber, S., Katus, H.A., Rottbauer, W., Meder, B., Hoheisel, J.D., Franke, A., and Meese, E. (2011b). Toward the blood-borne miRNome of human diseases. *Nat Methods* 8**,** 841-843.

Keller, A., Leidinger, P., Borries, A., Wendschlag, A., Wucherpfennig, F., Scheffler, M., Huwer, H., Lenhof, H.P., and Meese, E. (2009a). miRNAs in lung cancer - studying complex fingerprints in patient's blood cells by microarray experiments. *BMC Cancer* 9**,** 353.

Keller, A., Leidinger, P., Gislefoss, R., Haugen, A., Langseth, H., Staehler, P., Lenhof, H.P., and Meese, E. (2011c). Stable serum miRNA profiles as potential tool for non-invasive lung cancer diagnosis. *RNA Biol* 8**,** 506-516.

Keller, A., Leidinger, P., Lange, J., Borries, A., Schroers, H., Scheffler, M., Lenhof, H.P., Ruprecht, K., and Meese, E. (2009b). Multiple sclerosis: microRNA expression profiles accurately differentiate patients with relapsing-remitting disease from healthy controls. *PLoS One* 4**,** e7440.

Khella, H.W., Bakhet, M., Allo, G., Jewett, M.A., Girgis, A.H., Latif, A., Girgis, H., Von Both, I., Bjarnason, G.A., and Yousef, G.M. (2013). miR-192, miR-194 and miR-215: a convergent microRNA network suppressing tumor progression in renal cell carcinoma. *Carcinogenesis* 34**,** 2231-2239.

Kim, A.H., Reimers, M., Maher, B., Williamson, V., Mcmichael, O., Mcclay, J.L., Van Den Oord, E.J., Riley, B.P., Kendler, K.S., and Vladimirov, V.I. (2010a). MicroRNA expression profiling in the prefrontal cortex of individuals affected with schizophrenia and bipolar disorders. *Schizophr Res* 124**,** 183-191.

Kim, C.H., Kim, H.K., Rettig, R.L., Kim, J., Lee, E.T., Aprelikova, O., Choi, I.J., Munroe, D.J., and Green, J.E. (2011). miRNA signature associated with outcome of gastric cancer patients following chemotherapy. *BMC Med Genomics* 4**,** 79.

Kim, T.H., Kim, Y.K., Kwon, Y., Heo, J.H., Kang, H., Kim, G., and An, H.J. (2010b). Deregulation of miR-519a, 153, and 485-5p and its clinicopathological relevance in ovarian epithelial tumours. *Histopathology* 57**,** 734-743.

Kitano, M., Rahbari, R., Patterson, E.E., Steinberg, S.M., Prasad, N.B., Wang, Y., Zeiger, M.A., and Kebebew, E. (2012). Evaluation of candidate diagnostic microRNAs in thyroid fine-needle aspiration biopsy samples. *Thyroid* 22**,** 285-291.

Kuhn, D.E., Nuovo, G.J., Martin, M.M., Malana, G.E., Pleister, A.P., Jiang, J., Schmittgen, T.D., Terry, A.V., Jr., Gardiner, K., Head, E., Feldman, D.S., and Elton, T.S. (2008). Human chromosome 21-derived miRNAs are overexpressed in down syndrome brains and hearts. *Biochem Biophys Res Commun* 370**,** 473-477.

Kuhn, D.E., Nuovo, G.J., Terry, A.V., Jr., Martin, M.M., Malana, G.E., Sansom, S.E., Pleister, A.P., Beck, W.D., Head, E., Feldman, D.S., and Elton, T.S. (2010). Chromosome 21-derived microRNAs provide an etiological basis for aberrant protein expression in human Down syndrome brains. *J Biol Chem* 285**,** 1529-1543.

Lan, F.F., Wang, H., Chen, Y.C., Chan, C.Y., Ng, S.S., Li, K., Xie, D., He, M.L., Lin, M.C., and Kung, H.F. (2011). Hsa-let-7g inhibits proliferation of hepatocellular carcinoma cells by downregulation of c-Myc and upregulation of p16(INK4A). *Int J Cancer* 128**,** 319-331.

Lang, Q., and Ling, C. (2012). MiR-124 suppresses cell proliferation in hepatocellular carcinoma by targeting PIK3CA. *Biochem Biophys Res Commun* 426**,** 247-252.

Lau, P., Bossers, K., Janky, R., Salta, E., Frigerio, C.S., Barbash, S., Rothman, R., Sierksma, A.S., Thathiah, A., Greenberg, D., Papadopoulou, A.S., Achsel, T., Ayoubi, T., Soreq, H., Verhaagen, J., Swaab, D.F., Aerts, S., and De Strooper, B. (2013). Alteration of the microRNA network during the progression of Alzheimer's disease. *EMBO Mol Med* 5**,** 1613-1634.

Lee, J.H., Voortman, J., Dingemans, A.M., Voeller, D.M., Pham, T., Wang, Y., and Giaccone, G. (2011a). MicroRNA expression and clinical outcome of small cell lung cancer. *PLoS One* 6**,** e21300.

Lee, J.W., Park, Y.A., Choi, J.J., Lee, Y.Y., Kim, C.J., Choi, C., Kim, T.J., Lee, N.W., Kim, B.G., and Bae, D.S. (2011b). The expression of the miRNA-200 family in endometrial endometrioid carcinoma. *Gynecol Oncol* 120**,** 56-62.

Lee, S.T., Chu, K., Im, W.S., Yoon, H.J., Im, J.Y., Park, J.E., Park, K.H., Jung, K.H., Lee, S.K., Kim, M., and Roh, J.K. (2011c). Altered microRNA regulation in Huntington's disease models. *Exp Neurol* 227**,** 172-179.

Lee, S.T., Chu, K., Jung, K.H., Yoon, H.J., Jeon, D., Kang, K.M., Park, K.H., Bae, E.K., Kim, M., Lee, S.K., and Roh, J.K. (2010). MicroRNAs induced during ischemic preconditioning. *Stroke* 41**,** 1646-1651.

Leidinger, P., Keller, A., Borries, A., Reichrath, J., Rass, K., Jager, S.U., Lenhof, H.P., and Meese, E. (2010). High-throughput miRNA profiling of human melanoma blood samples. *BMC Cancer* 10**,** 262.

Lerman, G., Avivi, C., Mardoukh, C., Barzilai, A., Tessone, A., Gradus, B., Pavlotsky, F., Barshack, I., Polak-Charcon, S., Orenstein, A., Hornstein, E., Sidi, Y., and Avni, D. (2011). MiRNA expression in psoriatic skin: reciprocal regulation of hsa-miR-99a and IGF-1R. *PLoS One* 6**,** e20916.

Li, A., Omura, N., Hong, S.M., Vincent, A., Walter, K., Griffith, M., Borges, M., and Goggins, M. (2010). Pancreatic cancers epigenetically silence SIP1 and hypomethylate and overexpress miR-200a/200b in association with elevated circulating miR-200a and miR-200b levels. *Cancer Res* 70**,** 5226-5237.

Li, A., Yu, J., Kim, H., Wolfgang, C.L., Canto, M.I., Hruban, R.H., and Goggins, M. (2013a). MicroRNA array analysis finds elevated serum miR-1290 accurately distinguishes patients with low-stage pancreatic cancer from healthy and disease controls. *Clin Cancer Res* 19**,** 3600-3610.

Li, B.L., Lu, W., Lu, C., Qu, J.J., Yang, T.T., Yan, Q., and Wan, X.P. (2013b). CpG island hypermethylation-associated silencing of microRNAs promotes human endometrial cancer. *Cancer Cell Int* 13**,** 44.

Li, D., Chen, P., Li, X.Y., Zhang, L.Y., Xiong, W., Zhou, M., Xiao, L., Zeng, F., Li, X.L., Wu, M.H., and Li, G.Y. (2011). Grade-specific expression profiles of miRNAs/mRNAs and docking study in human grade I-III astrocytomas. *OMICS* 15**,** 673-682.

Li, H., Yin, C., Zhang, B., Sun, Y., Shi, L., Liu, N., Liang, S., Lu, S., Liu, Y., Zhang, J., Li, F., Li, W., Liu, F., Sun, L., and Qi, Y. (2013c). PTTG1 promotes migration and invasion of human non-small cell lung cancer cells and is modulated by miR-186. *Carcinogenesis* 34**,** 2145-2155.

Li, J., Du, L., Yang, Y., Wang, C., Liu, H., Wang, L., Zhang, X., Li, W., Zheng, G., and Dong, Z. (2013d). MiR-429 is an independent prognostic factor in colorectal cancer and exerts its anti-apoptotic function by targeting SOX2. *Cancer Lett* 329**,** 84-90.

Li, J.Y., Mu, D.L., Mu, L.H., Xin, M.Q., and Luan, J. (2013e). Functional annotation of the microRNA-mediated network in gigantomastia by integrating microRNA and mRNA expression profiling. *Chin Med J (Engl)* 126**,** 740-746.

Li, K.K., Pang, J.C., Ching, A.K., Wong, C.K., Kong, X., Wang, Y., Zhou, L., Chen, Z., and Ng, H.K. (2009). miR-124 is frequently down-regulated in medulloblastoma and is a negative regulator of SLC16A1. *Hum Pathol* 40**,** 1234-1243.

Li, S., Meng, H., Zhou, F., Zhai, L., Zhang, L., Gu, F., Fan, Y., Lang, R., Fu, L., Gu, L., and Qi, L. (2013f). MicroRNA-132 is frequently down-regulated in ductal carcinoma in situ (DCIS) of breast and acts as a tumor suppressor by inhibiting cell proliferation. *Pathol Res Pract* 209**,** 179-183.

Li, W., Xie, L., He, X., Li, J., Tu, K., Wei, L., Wu, J., Guo, Y., Ma, X., Zhang, P., Pan, Z., Hu, X., Zhao, Y., Xie, H., Jiang, G., Chen, T., Wang, J., Zheng, S., Cheng, J., Wan, D., Yang, S., Li, Y., and Gu, J. (2008). Diagnostic and prognostic implications of microRNAs in human hepatocellular carcinoma. *Int J Cancer* 123**,** 1616-1622.

Li, Z., Gu, X., Fang, Y., Xiang, J., and Chen, Z. (2012). microRNA expression profiles in human colorectal cancers with brain metastases. *Oncol Lett* 3**,** 346-350.

Lian, J., Zhang, X., Tian, H., Liang, N., Wang, Y., Liang, C., Li, X., and Sun, F. (2009). Altered microRNA expression in patients with non-obstructive azoospermia. *Reprod Biol Endocrinol* 7**,** 13.

Liang, D., Lin, X., and Lan, K. (2011a). Looking at Kaposi's Sarcoma-Associated Herpesvirus-Host Interactions from a microRNA Viewpoint. *Front Microbiol* 2**,** 271.

Liang, P., Lv, C., Jiang, B., Long, X., Zhang, P., Zhang, M., Xie, T., and Huang, X. (2012). MicroRNA profiling in denatured dermis of deep burn patients. *Burns* 38**,** 534-540.

Liang, S., He, L., Zhao, X., Miao, Y., Gu, Y., Guo, C., Xue, Z., Dou, W., Hu, F., Wu, K., Nie, Y., and Fan, D. (2011b). MicroRNA let-7f inhibits tumor invasion and metastasis by targeting MYH9 in human gastric cancer. *PLoS One* 6**,** e18409.

Liang, Y.J., Wang, Q.Y., Zhou, C.X., Yin, Q.Q., He, M., Yu, X.T., Cao, D.X., Chen, G.Q., He, J.R., and Zhao, Q. (2013). MiR-124 targets Slug to regulate epithelial-mesenchymal transition and metastasis of breast cancer. *Carcinogenesis* 34**,** 713-722.

Lin, J., Horikawa, Y., Tamboli, P., Clague, J., Wood, C.G., and Wu, X. (2010a). Genetic variations in microRNA-related genes are associated with survival and recurrence in patients with renal cell carcinoma. *Carcinogenesis* 31**,** 1805-1812.

Lin, J., Huang, S., Wu, S., Ding, J., Zhao, Y., Liang, L., Tian, Q., Zha, R., Zhan, R., and He, X. (2011). MicroRNA-423 promotes cell growth and regulates G(1)/S transition by targeting p21Cip1/Waf1 in hepatocellular carcinoma. *Carcinogenesis* 32**,** 1641-1647.

Lin, S.C., Liu, C.J., Lin, J.A., Chiang, W.F., Hung, P.S., and Chang, K.W. (2010b). miR-24 up-regulation in oral carcinoma: positive association from clinical and in vitro analysis. *Oral Oncol* 46**,** 204-208.

Liu, D.Z., Tian, Y., Ander, B.P., Xu, H., Stamova, B.S., Zhan, X., Turner, R.J., Jickling, G., and Sharp, F.R. (2010a). Brain and blood microRNA expression profiling of ischemic stroke, intracerebral hemorrhage, and kainate seizures. *J Cereb Blood Flow Metab* 30**,** 92-101.

Liu, H., Brannon, A.R., Reddy, A.R., Alexe, G., Seiler, M.W., Arreola, A., Oza, J.H., Yao, M., Juan, D., Liou, L.S., Ganesan, S., Levine, A.J., Rathmell, W.K., and Bhanot, G.V. (2010b). Identifying mRNA targets of microRNA dysregulated in cancer: with application to clear cell Renal Cell Carcinoma. *BMC Syst Biol* 4**,** 51.

Liu, J., Mao, Q., Liu, Y., Hao, X., Zhang, S., and Zhang, J. (2013a). Analysis of miR-205 and miR-155 expression in the blood of breast cancer patients. *Chin J Cancer Res* 25**,** 46-54.

Liu, K., Zhao, H., Yao, H., Lei, S., Lei, Z., Li, T., and Qi, H. (2013b). MicroRNA-124 regulates the proliferation of colorectal cancer cells by targeting iASPP. *Biomed Res Int* 2013**,** 867537.

Liu, R., Zhang, C., Hu, Z., Li, G., Wang, C., Yang, C., Huang, D., Chen, X., Zhang, H., Zhuang, R., Deng, T., Liu, H., Yin, J., Wang, S., Zen, K., Ba, Y., and Zhang, C.Y. (2011). A five-microRNA signature identified from genome-wide serum microRNA expression profiling serves as a fingerprint for gastric cancer diagnosis. *Eur J Cancer* 47**,** 784-791.

Liu, T., Cheng, W., Gao, Y., Wang, H., and Liu, Z. (2012a). Microarray analysis of microRNA expression patterns in the semen of infertile men with semen abnormalities. *Mol Med Rep* 6**,** 535-542.

Liu, X.G., Zhu, W.Y., Huang, Y.Y., Ma, L.N., Zhou, S.Q., Wang, Y.K., Zeng, F., Zhou, J.H., and Zhang, Y.K. (2012b). High expression of serum miR-21 and tumor miR-200c associated with poor prognosis in patients with lung cancer. *Med Oncol* 29**,** 618-626.

Liu, Y., Hei, Y., Shu, Q., Dong, J., Gao, Y., Fu, H., Zheng, X., and Yang, G. (2012c). VCP/p97, down-regulated by microRNA-129-5p, could regulate the progression of hepatocellular carcinoma. *PLoS One* 7**,** e35800.

Long, C., Jiang, L., Wei, F., Ma, C., Zhou, H., Yang, S., Liu, X., and Liu, Z. (2013). Integrated miRNA-mRNA analysis revealing the potential roles of miRNAs in chordomas. *PLoS One* 8**,** e66676.

Lu, J., Kwan, B.C., Lai, F.M., Tam, L.S., Li, E.K., Chow, K.M., Wang, G., Li, P.K., and Szeto, C.C. (2012a). Glomerular and tubulointerstitial miR-638, miR-198 and miR-146a expression in lupus nephritis. *Nephrology (Carlton)* 17**,** 346-351.

Lu, T.X., Sherrill, J.D., Wen, T., Plassard, A.J., Besse, J.A., Abonia, J.P., Franciosi, J.P., Putnam, P.E., Eby, M., Martin, L.J., Aronow, B.J., and Rothenberg, M.E. (2012b). MicroRNA signature in patients with eosinophilic esophagitis, reversibility with glucocorticoids, and assessment as disease biomarkers. *J Allergy Clin Immunol* 129**,** 1064-1075 e1069.

Lukiw, W.J., and Alexandrov, P.N. (2012). Regulation of complement factor H (CFH) by multiple miRNAs in Alzheimer's disease (AD) brain. *Mol Neurobiol* 46**,** 11-19.

Lungu, G., Stoica, G., and Ambrus, A. (2013). MicroRNA profiling and the role of microRNA-132 in neurodegeneration using a rat model. *Neurosci Lett* 553**,** 153-158.

Luo, X., Zhang, L., Li, M., Zhang, W., Leng, X., Zhang, F., Zhao, Y., and Zeng, X. (2013). The role of miR-125b in T lymphocytes in the pathogenesis of systemic lupus erythematosus. *Clin Exp Rheumatol* 31**,** 263-271.

Luo, Z., Zhang, L., Li, Z., Li, X., Li, G., Yu, H., Jiang, C., Dai, Y., Guo, X., Xiang, J., and Li, G. (2012). An in silico analysis of dynamic changes in microRNA expression profiles in stepwise development of nasopharyngeal carcinoma. *BMC Med Genomics* 5**,** 3.

Lusardi, T.A., Farr, C.D., Faulkner, C.L., Pignataro, G., Yang, T., Lan, J., Simon, R.P., and Saugstad, J.A. (2010). Ischemic preconditioning regulates expression of microRNAs and a predicted target, MeCP2, in mouse cortex. *J Cereb Blood Flow Metab* 30**,** 744-756.

Lussier, Y.A., Khodarev, N.N., Regan, K., Corbin, K., Li, H., Ganai, S., Khan, S.A., Gnerlich, J.L., Darga, T.E., Fan, H., Karpenko, O., Paty, P.B., Posner, M.C., Chmura, S.J., Hellman, S., Ferguson, M.K., and Weichselbaum, R.R. (2012). Oligo- and polymetastatic progression in lung metastasis(es) patients is associated with specific microRNAs. *PLoS One* 7**,** e50141.

Lv, X.B., Jiao, Y., Qing, Y., Hu, H., Cui, X., Lin, T., Song, E., and Yu, F. (2011). miR-124 suppresses multiple steps of breast cancer metastasis by targeting a cohort of pro-metastatic genes in vitro. *Chin J Cancer* 30**,** 821-830.

Madhavan, D., Zucknick, M., Wallwiener, M., Cuk, K., Modugno, C., Scharpff, M., Schott, S., Heil, J., Turchinovich, A., Yang, R., Benner, A., Riethdorf, S., Trumpp, A., Sohn, C., Pantel, K., Schneeweiss, A., and Burwinkel, B. (2012). Circulating miRNAs as surrogate markers for circulating tumor cells and prognostic markers in metastatic breast cancer. *Clin Cancer Res* 18**,** 5972-5982.

Maharshak, N., Shenhar-Tsarfaty, S., Aroyo, N., Orpaz, N., Guberman, I., Canaani, J., Halpern, Z., Dotan, I., Berliner, S., and Soreq, H. (2013). MicroRNA-132 modulates cholinergic signaling and inflammation in human inflammatory bowel disease. *Inflamm Bowel Dis* 19**,** 1346-1353.

Manca, S., Magrelli, A., Cialfi, S., Lefort, K., Ambra, R., Alimandi, M., Biolcati, G., Uccelletti, D., Palleschi, C., Screpanti, I., Candi, E., Melino, G., Salvatore, M., Taruscio, D., and Talora, C. (2011). Oxidative stress activation of miR-125b is part of the molecular switch for Hailey-Hailey disease manifestation. *Exp Dermatol* 20**,** 932-937.

Mar-Aguilar, F., Luna-Aguirre, C.M., Moreno-Rocha, J.C., Araiza-Chavez, J., Trevino, V., Rodriguez-Padilla, C., and Resendez-Perez, D. (2013). Differential expression of miR-21, miR-125b and miR-191 in breast cancer tissue. *Asia Pac J Clin Oncol* 9**,** 53-59.

Marchini, S., Cavalieri, D., Fruscio, R., Calura, E., Garavaglia, D., Nerini, I.F., Mangioni, C., Cattoretti, G., Clivio, L., Beltrame, L., Katsaros, D., Scarampi, L., Menato, G., Perego, P., Chiorino, G., Buda, A., Romualdi, C., and D'incalci, M. (2011). Association between miR-200c and the survival of patients with stage I epithelial ovarian cancer: a retrospective study of two independent tumour tissue collections. *Lancet Oncol* 12**,** 273-285.

Marques, F.Z., Campain, A.E., Tomaszewski, M., Zukowska-Szczechowska, E., Yang, Y.H., Charchar, F.J., and Morris, B.J. (2011). Gene expression profiling reveals renin mRNA overexpression in human hypertensive kidneys and a role for microRNAs. *Hypertension* 58**,** 1093-1098.

Matsukawa, T., Sakai, T., Yonezawa, T., Hiraiwa, H., Hamada, T., Nakashima, M., Ono, Y., Ishizuka, S., Nakahara, H., Lotz, M.K., Asahara, H., and Ishiguro, N. (2013). MicroRNA-125b regulates the expression of aggrecanase-1 (ADAMTS-4) in human osteoarthritic chondrocytes. *Arthritis Res Ther* 15**,** R28.

Matsumoto, S., Sakata, Y., Suna, S., Nakatani, D., Usami, M., Hara, M., Kitamura, T., Hamasaki, T., Nanto, S., Kawahara, Y., and Komuro, I. (2013). Circulating p53-responsive microRNAs are predictive indicators of heart failure after acute myocardial infarction. *Circ Res* 113**,** 322-326.

Mcarthur, K., Feng, B., Wu, Y., Chen, S., and Chakrabarti, S. (2011). MicroRNA-200b regulates vascular endothelial growth factor-mediated alterations in diabetic retinopathy. *Diabetes* 60**,** 1314-1323.

Meder, B., Keller, A., Vogel, B., Haas, J., Sedaghat-Hamedani, F., Kayvanpour, E., Just, S., Borries, A., Rudloff, J., Leidinger, P., Meese, E., Katus, H.A., and Rottbauer, W. (2011). MicroRNA signatures in total peripheral blood as novel biomarkers for acute myocardial infarction. *Basic Res Cardiol* 106**,** 13-23.

Meerson, A., Traurig, M., Ossowski, V., Fleming, J.M., Mullins, M., and Baier, L.J. (2013). Human adipose microRNA-221 is upregulated in obesity and affects fat metabolism downstream of leptin and TNF-alpha. *Diabetologia* 56**,** 1971-1979.

Mees, S.T., Mardin, W.A., Wendel, C., Baeumer, N., Willscher, E., Senninger, N., Schleicher, C., Colombo-Benkmann, M., and Haier, J. (2010). EP300--a miRNA-regulated metastasis suppressor gene in ductal adenocarcinomas of the pancreas. *Int J Cancer* 126**,** 114-124.

Miller, B.H., Zeier, Z., Xi, L., Lanz, T.A., Deng, S., Strathmann, J., Willoughby, D., Kenny, P.J., Elsworth, J.D., Lawrence, M.S., Roth, R.H., Edbauer, D., Kleiman, R.J., and Wahlestedt, C. (2012). MicroRNA-132 dysregulation in schizophrenia has implications for both neurodevelopment and adult brain function. *Proc Natl Acad Sci U S A* 109**,** 3125-3130.

Mizuno, S., Bogaard, H.J., Gomez-Arroyo, J., Alhussaini, A., Kraskauskas, D., Cool, C.D., and Voelkel, N.F. (2012). MicroRNA-199a-5p is associated with hypoxia-inducible factor-1alpha expression in lungs from patients with COPD. *Chest* 142**,** 663-672.

Muinos-Gimeno, M., Guidi, M., Kagerbauer, B., Martin-Santos, R., Navines, R., Alonso, P., Menchon, J.M., Gratacos, M., Estivill, X., and Espinosa-Parrilla, Y. (2009). Allele variants in functional MicroRNA target sites of the neurotrophin-3 receptor gene (NTRK3) as susceptibility factors for anxiety disorders. *Hum Mutat* 30**,** 1062-1071.

Murata, K., Furu, M., Yoshitomi, H., Ishikawa, M., Shibuya, H., Hashimoto, M., Imura, Y., Fujii, T., Ito, H., Mimori, T., and Matsuda, S. (2013). Comprehensive microRNA analysis identifies miR-24 and miR-125a-5p as plasma biomarkers for rheumatoid arthritis. *PLoS One* 8**,** e69118.

Murata, K., Yoshitomi, H., Tanida, S., Ishikawa, M., Nishitani, K., Ito, H., and Nakamura, T. (2010). Plasma and synovial fluid microRNAs as potential biomarkers of rheumatoid arthritis and osteoarthritis. *Arthritis Res Ther* 12**,** R86.

Nakada, C., Matsuura, K., Tsukamoto, Y., Tanigawa, M., Yoshimoto, T., Narimatsu, T., Nguyen, L.T., Hijiya, N., Uchida, T., Sato, F., Mimata, H., Seto, M., and Moriyama, M. (2008). Genome-wide microRNA expression profiling in renal cell carcinoma: significant down-regulation of miR-141 and miR-200c. *J Pathol* 216**,** 418-427.

Nakashima, T., Jinnin, M., Yamane, K., Honda, N., Kajihara, I., Makino, T., Masuguchi, S., Fukushima, S., Okamoto, Y., Hasegawa, M., Fujimoto, M., and Ihn, H. (2012). Impaired IL-17 signaling pathway contributes to the increased collagen expression in scleroderma fibroblasts. *J Immunol* 188**,** 3573-3583.

Nam, E.J., Yoon, H., Kim, S.W., Kim, H., Kim, Y.T., Kim, J.H., Kim, J.W., and Kim, S. (2008). MicroRNA expression profiles in serous ovarian carcinoma. *Clin Cancer Res* 14**,** 2690-2695.

Nesca, V., Guay, C., Jacovetti, C., Menoud, V., Peyot, M.L., Laybutt, D.R., Prentki, M., and Regazzi, R. (2013). Identification of particular groups of microRNAs that positively or negatively impact on beta cell function in obese models of type 2 diabetes. *Diabetologia* 56**,** 2203-2212.

Ni, C.W., Qiu, H., and Jo, H. (2011). MicroRNA-663 upregulated by oscillatory shear stress plays a role in inflammatory response of endothelial cells. *Am J Physiol Heart Circ Physiol* 300**,** H1762-1769.

Nishida, N., Nagahara, M., Sato, T., Mimori, K., Sudo, T., Tanaka, F., Shibata, K., Ishii, H., Sugihara, K., Doki, Y., and Mori, M. (2012). Microarray analysis of colorectal cancer stromal tissue reveals upregulation of two oncogenic miRNA clusters. *Clin Cancer Res* 18**,** 3054-3070.

Nishida, N., Yokobori, T., Mimori, K., Sudo, T., Tanaka, F., Shibata, K., Ishii, H., Doki, Y., Kuwano, H., and Mori, M. (2011). MicroRNA miR-125b is a prognostic marker in human colorectal cancer. *Int J Oncol* 38**,** 1437-1443.

Niyazi, M., Zehentmayr, F., Niemoller, O.M., Eigenbrod, S., Kretzschmar, H., Schulze-Osthoff, K., Tonn, J.C., Atkinson, M., Mortl, S., and Belka, C. (2011). MiRNA expression patterns predict survival in glioblastoma. *Radiat Oncol* 6**,** 153.

Nymark, P., Guled, M., Borze, I., Faisal, A., Lahti, L., Salmenkivi, K., Kettunen, E., Anttila, S., and Knuutila, S. (2011). Integrative analysis of microRNA, mRNA and aCGH data reveals asbestos- and histology-related changes in lung cancer. *Genes Chromosomes Cancer* 50**,** 585-597.

Oak, S.R., Murray, L., Herath, A., Sleeman, M., Anderson, I., Joshi, A.D., Coelho, A.L., Flaherty, K.R., Toews, G.B., Knight, D., Martinez, F.J., and Hogaboam, C.M. (2011). A micro RNA processing defect in rapidly progressing idiopathic pulmonary fibrosis. *PLoS One* 6**,** e21253.

Odar, K., Bostjancic, E., Gale, N., Glavac, D., and Zidar, N. (2012). Differential expression of microRNAs miR-21, miR-31, miR-203, miR-125a-5p and miR-125b and proteins PTEN and p63 in verrucous carcinoma of the head and neck. *Histopathology* 61**,** 257-265.

Ogawa, T., Enomoto, M., Fujii, H., Sekiya, Y., Yoshizato, K., Ikeda, K., and Kawada, N. (2012a). MicroRNA-221/222 upregulation indicates the activation of stellate cells and the progression of liver fibrosis. *Gut* 61**,** 1600-1609.

Ogawa, T., Saiki, Y., Shiga, K., Chen, N., Fukushige, S., Sunamura, M., Nagase, H., Hashimoto, S., Matsuura, K., Saijo, S., Kobayashi, T., and Horii, A. (2012b). miR-34a is downregulated in cis-diamminedichloroplatinum treated sinonasal squamous cell carcinoma patients with poor prognosis. *Cancer Sci* 103**,** 1737-1743.

Ohlsson Teague, E.M., Van Der Hoek, K.H., Van Der Hoek, M.B., Perry, N., Wagaarachchi, P., Robertson, S.A., Print, C.G., and Hull, L.M. (2009). MicroRNA-regulated pathways associated with endometriosis. *Mol Endocrinol* 23**,** 265-275.

Oliveira-Carvalho, V., Da Silva, M.M., Guimaraes, G.V., Bacal, F., and Bocchi, E.A. (2013). MicroRNAs: new players in heart failure. *Mol Biol Rep* 40**,** 2663-2670.

Ortega, F.J., Mercader, J.M., Catalan, V., Moreno-Navarrete, J.M., Pueyo, N., Sabater, M., Gomez-Ambrosi, J., Anglada, R., Fernandez-Formoso, J.A., Ricart, W., Fruhbeck, G., and Fernandez-Real, J.M. (2013). Targeting the circulating microRNA signature of obesity. *Clin Chem* 59**,** 781-792.

Oshikawa, Y., Jinnin, M., Makino, T., Kajihara, I., Makino, K., Honda, N., Nakayama, W., Inoue, K., Fukushima, S., and Ihn, H. (2013). Decreased miR-7 expression in the skin and sera of patients with dermatomyositis. *Acta Derm Venereol* 93**,** 273-276.

Oster, B., Linnet, L., Christensen, L.L., Thorsen, K., Ongen, H., Dermitzakis, E.T., Sandoval, J., Moran, S., Esteller, M., Hansen, T.F., Lamy, P., Group, C.S., Laurberg, S., Orntoft, T.F., and Andersen, C.L. (2013). Non-CpG island promoter hypomethylation and miR-149 regulate the expression of SRPX2 in colorectal cancer. *Int J Cancer* 132**,** 2303-2315.

Ozata, D.M., Caramuta, S., Velazquez-Fernandez, D., Akcakaya, P., Xie, H., Hoog, A., Zedenius, J., Backdahl, M., Larsson, C., and Lui, W.O. (2011). The role of microRNA deregulation in the pathogenesis of adrenocortical carcinoma. *Endocr Relat Cancer* 18**,** 643-655.

Ozsait, B., Komurcu-Bayrak, E., Levula, M., Erginel-Unaltuna, N., Kahonen, M., Rai, M., Lehtimaki, T., and Laaksonen, R. (2010). Niemann-Pick type C fibroblasts have a distinct microRNA profile related to lipid metabolism and certain cellular components. *Biochem Biophys Res Commun* 403**,** 316-321.

Packer, A.N., Xing, Y., Harper, S.Q., Jones, L., and Davidson, B.L. (2008). The bifunctional microRNA miR-9/miR-9* regulates REST and CoREST and is downregulated in Huntington's disease. *J Neurosci* 28**,** 14341-14346.

Padgett, K.A., Lan, R.Y., Leung, P.C., Lleo, A., Dawson, K., Pfeiff, J., Mao, T.K., Coppel, R.L., Ansari, A.A., and Gershwin, M.E. (2009). Primary biliary cirrhosis is associated with altered hepatic microRNA expression. *J Autoimmun* 32**,** 246-253.

Paraskevi, A., Theodoropoulos, G., Papaconstantinou, I., Mantzaris, G., Nikiteas, N., and Gazouli, M. (2012). Circulating MicroRNA in inflammatory bowel disease. *J Crohns Colitis* 6**,** 900-904.

Park, J.K., Henry, J.C., Jiang, J., Esau, C., Gusev, Y., Lerner, M.R., Postier, R.G., Brackett, D.J., and Schmittgen, T.D. (2011). miR-132 and miR-212 are increased in pancreatic cancer and target the retinoblastoma tumor suppressor. *Biochem Biophys Res Commun* 406**,** 518-523.

Peng, J., Omran, A., Ashhab, M.U., Kong, H., Gan, N., He, F., and Yin, F. (2013). Expression patterns of miR-124, miR-134, miR-132, and miR-21 in an immature rat model and children with mesial temporal lobe epilepsy. *J Mol Neurosci* 50**,** 291-297.

Perkins, D.O., Jeffries, C.D., Jarskog, L.F., Thomson, J.M., Woods, K., Newman, M.A., Parker, J.S., Jin, J., and Hammond, S.M. (2007). microRNA expression in the prefrontal cortex of individuals with schizophrenia and schizoaffective disorder. *Genome Biol* 8**,** R27.

Pignot, G., Cizeron-Clairac, G., Vacher, S., Susini, A., Tozlu, S., Vieillefond, A., Zerbib, M., Lidereau, R., Debre, B., Amsellem-Ouazana, D., and Bieche, I. (2013). microRNA expression profile in a large series of bladder tumors: identification of a 3-miRNA signature associated with aggressiveness of muscle-invasive bladder cancer. *Int J Cancer* 132**,** 2479-2491.

Qin, L., Chen, Y., Niu, Y., Chen, W., Wang, Q., Xiao, S., Li, A., Xie, Y., Li, J., Zhao, X., He, Z., and Mo, D. (2010). A deep investigation into the adipogenesis mechanism: profile of microRNAs regulating adipogenesis by modulating the canonical Wnt/beta-catenin signaling pathway. *BMC Genomics* 11**,** 320.

Ranade, A.R., Cherba, D., Sridhar, S., Richardson, P., Webb, C., Paripati, A., Bowles, B., and Weiss, G.J. (2010). MicroRNA 92a-2*: a biomarker predictive for chemoresistance and prognostic for survival in patients with small cell lung cancer. *J Thorac Oncol* 5**,** 1273-1278.

Reddy, M.A., Jin, W., Villeneuve, L., Wang, M., Lanting, L., Todorov, I., Kato, M., and Natarajan, R. (2012). Pro-inflammatory role of microrna-200 in vascular smooth muscle cells from diabetic mice. *Arterioscler Thromb Vasc Biol* 32**,** 721-729.

Redell, J.B., Moore, A.N., Ward, N.H., 3rd, Hergenroeder, G.W., and Dash, P.K. (2010). Human traumatic brain injury alters plasma microRNA levels. *J Neurotrauma* 27**,** 2147-2156.

Reijerkerk, A., Lopez-Ramirez, M.A., Van Het Hof, B., Drexhage, J.A., Kamphuis, W.W., Kooij, G., Vos, J.B., Van Der Pouw Kraan, T.C., Van Zonneveld, A.J., Horrevoets, A.J., Prat, A., Romero, I.A., and De Vries, H.E. (2013). MicroRNAs regulate human brain endothelial cell-barrier function in inflammation: implications for multiple sclerosis. *J Neurosci* 33**,** 6857-6863.

Rink, C., and Khanna, S. (2011). MicroRNA in ischemic stroke etiology and pathology. *Physiol Genomics* 43**,** 521-528.

Rio-Machin, A., Ferreira, B.I., Henry, T., Gomez-Lopez, G., Agirre, X., Alvarez, S., Rodriguez-Perales, S., Prosper, F., Calasanz, M.J., Martinez, J., Fonseca, R., and Cigudosa, J.C. (2013). Downregulation of specific miRNAs in hyperdiploid multiple myeloma mimics the oncogenic effect of IgH translocations occurring in the non-hyperdiploid subtype. *Leukemia* 27**,** 925-931.

Rossing, M., Borup, R., Henao, R., Winther, O., Vikesaa, J., Niazi, O., Godballe, C., Krogdahl, A., Glud, M., Hjort-Sorensen, C., Kiss, K., Bennedbaek, F.N., and Nielsen, F.C. (2012). Down-regulation of microRNAs controlling tumourigenic factors in follicular thyroid carcinoma. *J Mol Endocrinol* 48**,** 11-23.

Ryan, B.M., Mcclary, A.C., Valeri, N., Robinson, D., Paone, A., Bowman, E.D., Robles, A.I., Croce, C., and Harris, C.C. (2012). rs4919510 in hsa-mir-608 is associated with outcome but not risk of colorectal cancer. *PLoS One* 7**,** e36306.

Saad, R., Chen, Z., Zhu, S., Jia, P., Zhao, Z., Washington, M.K., Belkhiri, A., and El-Rifai, W. (2013). Deciphering the unique microRNA signature in human esophageal adenocarcinoma. *PLoS One* 8**,** e64463.

Salvi, A., Abeni, E., Portolani, N., Barlati, S., and De Petro, G. (2013). Human hepatocellular carcinoma cell-specific miRNAs reveal the differential expression of miR-24 and miR-27a in cirrhotic/non-cirrhotic HCC. *Int J Oncol* 42**,** 391-402.

Sand, M., Sand, D., Altmeyer, P., and Bechara, F.G. (2012a). MicroRNA in non-melanoma skin cancer. *Cancer Biomark* 11**,** 253-257.

Sand, M., Skrygan, M., Sand, D., Georgas, D., Gambichler, T., Hahn, S.A., Altmeyer, P., and Bechara, F.G. (2013). Comparative microarray analysis of microRNA expression profiles in primary cutaneous malignant melanoma, cutaneous malignant melanoma metastases, and benign melanocytic nevi. *Cell Tissue Res* 351**,** 85-98.

Sand, M., Skrygan, M., Sand, D., Georgas, D., Hahn, S.A., Gambichler, T., Altmeyer, P., and Bechara, F.G. (2012b). Expression of microRNAs in basal cell carcinoma. *Br J Dermatol* 167**,** 847-855.

Santini, P., Politi, L., Vedova, P.D., Scandurra, R., and Scotto D'abusco, A. (2013). The inflammatory circuitry of miR-149 as a pathological mechanism in osteoarthritis. *Rheumatol Int*.

Saydam, O., Senol, O., Wurdinger, T., Mizrak, A., Ozdener, G.B., Stemmer-Rachamimov, A.O., Yi, M., Stephens, R.M., Krichevsky, A.M., Saydam, N., Brenner, G.J., and Breakefield, X.O. (2011). miRNA-7 attenuation in Schwannoma tumors stimulates growth by upregulating three oncogenic signaling pathways. *Cancer Res* 71**,** 852-861.

Schaefer, A., Jung, M., Mollenkopf, H.J., Wagner, I., Stephan, C., Jentzmik, F., Miller, K., Lein, M., Kristiansen, G., and Jung, K. (2010). Diagnostic and prognostic implications of microRNA profiling in prostate carcinoma. *Int J Cancer* 126**,** 1166-1176.

Scheffer, A.R., Holdenrieder, S., Kristiansen, G., Von Ruecker, A., Muller, S.C., and Ellinger, J. (2012). Circulating microRNAs in serum: novel biomarkers for patients with bladder cancer? *World J Urol*.

Schipper, H.M., Maes, O.C., Chertkow, H.M., and Wang, E. (2007). MicroRNA expression in Alzheimer blood mononuclear cells. *Gene Regul Syst Bio* 1**,** 263-274.

Schmitz, K.J., Helwig, J., Bertram, S., Sheu, S.Y., Suttorp, A.C., Seggewiss, J., Willscher, E., Walz, M.K., Worm, K., and Schmid, K.W. (2011). Differential expression of microRNA-675, microRNA-139-3p and microRNA-335 in benign and malignant adrenocortical tumours. *J Clin Pathol* 64**,** 529-535.

Schultz, N.A., Andersen, K.K., Roslind, A., Willenbrock, H., Wojdemann, M., and Johansen, J.S. (2012a). Prognostic microRNAs in cancer tissue from patients operated for pancreatic cancer--five microRNAs in a prognostic index. *World J Surg* 36**,** 2699-2707.

Schultz, N.A., Werner, J., Willenbrock, H., Roslind, A., Giese, N., Horn, T., Wojdemann, M., and Johansen, J.S. (2012b). MicroRNA expression profiles associated with pancreatic adenocarcinoma and ampullary adenocarcinoma. *Mod Pathol* 25**,** 1609-1622.

Selth, L.A., Townley, S., Gillis, J.L., Ochnik, A.M., Murti, K., Macfarlane, R.J., Chi, K.N., Marshall, V.R., Tilley, W.D., and Butler, L.M. (2012). Discovery of circulating microRNAs associated with human prostate cancer using a mouse model of disease. *Int J Cancer* 131**,** 652-661.

Senanayake, U., Das, S., Vesely, P., Alzoughbi, W., Frohlich, L.F., Chowdhury, P., Leuschner, I., Hoefler, G., and Guertl, B. (2012). miR-192, miR-194, miR-215, miR-200c and miR-141 are downregulated and their common target ACVR2B is strongly expressed in renal childhood neoplasms. *Carcinogenesis* 33**,** 1014-1021.

Shah, A.A., Leidinger, P., Backes, C., Keller, A., Karpinski, P., Sasiadek, M.M., Blin, N., and Meese, E. (2013). A set of specific miRNAs is connected with murine and human gastric cancer. *Genes Chromosomes Cancer* 52**,** 237-249.

Shaked, I., Meerson, A., Wolf, Y., Avni, R., Greenberg, D., Gilboa-Geffen, A., and Soreq, H. (2009). MicroRNA-132 potentiates cholinergic anti-inflammatory signaling by targeting acetylcholinesterase. *Immunity* 31**,** 965-973.

Shaltiel, G., Hanan, M., Wolf, Y., Barbash, S., Kovalev, E., Shoham, S., and Soreq, H. (2013). Hippocampal microRNA-132 mediates stress-inducible cognitive deficits through its acetylcholinesterase target. *Brain Struct Funct* 218**,** 59-72.

Shang, C., Lu, Y.M., and Meng, L.R. (2012). MicroRNA-125b down-regulation mediates endometrial cancer invasion by targeting ERBB2. *Med Sci Monit* 18**,** BR149-155.

Shaoqing, Y., Ruxin, Z., Guojun, L., Zhiqiang, Y., Hua, H., Shudong, Y., and Jie, Z. (2011). Microarray analysis of differentially expressed microRNAs in allergic rhinitis. *Am J Rhinol Allergy* 25**,** e242-246.

Sheinerman, K.S., Tsivinsky, V.G., Crawford, F., Mullan, M.J., Abdullah, L., and Umansky, S.R. (2012). Plasma microRNA biomarkers for detection of mild cognitive impairment. *Aging (Albany NY)* 4**,** 590-605.

Shen, Q., Cicinnati, V.R., Zhang, X., Iacob, S., Weber, F., Sotiropoulos, G.C., Radtke, A., Lu, M., Paul, A., Gerken, G., and Beckebaum, S. (2010). Role of microRNA-199a-5p and discoidin domain receptor 1 in human hepatocellular carcinoma invasion. *Mol Cancer* 9**,** 227.

Shi, X.B., Xue, L., Ma, A.H., Tepper, C.G., Gandour-Edwards, R., Kung, H.J., and Devere White, R.W. (2013). Tumor suppressive miR-124 targets androgen receptor and inhibits proliferation of prostate cancer cells. *Oncogene* 32**,** 4130-4138.

Shiiba, M., Shinozuka, K., Saito, K., Fushimi, K., Kasamatsu, A., Ogawara, K., Uzawa, K., Ito, H., Takiguchi, Y., and Tanzawa, H. (2013). MicroRNA-125b regulates proliferation and radioresistance of oral squamous cell carcinoma. *Br J Cancer* 108**,** 1817-1821.

Shimizu, T., Suzuki, H., Nojima, M., Kitamura, H., Yamamoto, E., Maruyama, R., Ashida, M., Hatahira, T., Kai, M., Masumori, N., Tokino, T., Imai, K., Tsukamoto, T., and Toyota, M. (2013). Methylation of a panel of microRNA genes is a novel biomarker for detection of bladder cancer. *Eur Urol* 63**,** 1091-1100.

Shinozaki, A., Sakatani, T., Ushiku, T., Hino, R., Isogai, M., Ishikawa, S., Uozaki, H., Takada, K., and Fukayama, M. (2010). Downregulation of microRNA-200 in EBV-associated gastric carcinoma. *Cancer Res* 70**,** 4719-4727.

Shwetha, S., Gouthamchandra, K., Chandra, M., Ravishankar, B., Khaja, M.N., and Das, S. (2013). Circulating miRNA profile in HCV infected serum: novel insight into pathogenesis. *Sci Rep* 3**,** 1555.

Silber, J., Hashizume, R., Felix, T., Hariono, S., Yu, M., Berger, M.S., Huse, J.T., Vandenberg, S.R., James, C.D., Hodgson, J.G., and Gupta, N. (2013). Expression of miR-124 inhibits growth of medulloblastoma cells. *Neuro Oncol* 15**,** 83-90.

Silber, J., Lim, D.A., Petritsch, C., Persson, A.I., Maunakea, A.K., Yu, M., Vandenberg, S.R., Ginzinger, D.G., James, C.D., Costello, J.F., Bergers, G., Weiss, W.A., Alvarez-Buylla, A., and Hodgson, J.G. (2008). miR-124 and miR-137 inhibit proliferation of glioblastoma multiforme cells and induce differentiation of brain tumor stem cells. *BMC Med* 6**,** 14.

Sinha, M., Ghose, J., and Bhattarcharyya, N.P. (2011). Micro RNA -214,-150,-146a and-125b target Huntingtin gene. *RNA Biol* 8**,** 1005-1021.

Skalsky, R.L., and Cullen, B.R. (2011). Reduced expression of brain-enriched microRNAs in glioblastomas permits targeted regulation of a cell death gene. *PLoS One* 6**,** e24248.

Slaby, O., Redova, M., Poprach, A., Nekvindova, J., Iliev, R., Radova, L., Lakomy, R., Svoboda, M., and Vyzula, R. (2012). Identification of MicroRNAs associated with early relapse after nephrectomy in renal cell carcinoma patients. *Genes Chromosomes Cancer* 51**,** 707-716.

Slattery, M.L., Wolff, E., Hoffman, M.D., Pellatt, D.F., Milash, B., and Wolff, R.K. (2011). MicroRNAs and colon and rectal cancer: differential expression by tumor location and subtype. *Genes Chromosomes Cancer* 50**,** 196-206.

Snowdon, J., Boag, S., Feilotter, H., Izard, J., and Siemens, D.R. (2012). A pilot study of urinary microRNA as a biomarker for urothelial cancer. *Can Urol Assoc J***,** 1-5.

Song, T., Xia, W., Shao, N., Zhang, X., Wang, C., Wu, Y., Dong, J., Cai, W., and Li, H. (2010). Differential miRNA expression profiles in bladder urothelial carcinomas. *Asian Pac J Cancer Prev* 11**,** 905-911.

Song, Y., Zhao, F., Wang, Z., Liu, Z., Chiang, Y., Xu, Y., Gao, P., and Xu, H. (2012). Inverse association between miR-194 expression and tumor invasion in gastric cancer. *Ann Surg Oncol* 19 Suppl 3**,** S509-517.

Sonntag, K.C., Woo, T.U., and Krichevsky, A.M. (2012). Converging miRNA functions in diverse brain disorders: a case for miR-124 and miR-126. *Exp Neurol* 235**,** 427-435.

Stark, M.S., Tyagi, S., Nancarrow, D.J., Boyle, G.M., Cook, A.L., Whiteman, D.C., Parsons, P.G., Schmidt, C., Sturm, R.A., and Hayward, N.K. (2010). Characterization of the Melanoma miRNAome by Deep Sequencing. *PLoS One* 5**,** e9685.

Sun, Y., Gui, H., Li, Q., Luo, Z.M., Zheng, M.J., Duan, J.L., and Liu, X. (2013). MicroRNA-124 protects neurons against apoptosis in cerebral ischemic stroke. *CNS Neurosci Ther* 19**,** 813-819.

Sun, Y., Zhao, X., Zhou, Y., and Hu, Y. (2012). miR-124, miR-137 and miR-340 regulate colorectal cancer growth via inhibition of the Warburg effect. *Oncol Rep* 28**,** 1346-1352.

Sundaram, P., Hultine, S., Smith, L.M., Dews, M., Fox, J.L., Biyashev, D., Schelter, J.M., Huang, Q., Cleary, M.A., Volpert, O.V., and Thomas-Tikhonenko, A. (2011). p53-responsive miR-194 inhibits thrombospondin-1 and promotes angiogenesis in colon cancers. *Cancer Res* 71**,** 7490-7501.

Szczyrba, J., Loprich, E., Wach, S., Jung, V., Unteregger, G., Barth, S., Grobholz, R., Wieland, W., Stohr, R., Hartmann, A., Wullich, B., and Grasser, F. (2010). The microRNA profile of prostate carcinoma obtained by deep sequencing. *Mol Cancer Res* 8**,** 529-538.

Takagi, T., Naito, Y., Mizushima, K., Hirata, I., Yagi, N., Tomatsuri, N., Ando, T., Oyamada, Y., Isozaki, Y., Hongo, H., Uchiyama, K., Handa, O., Kokura, S., Ichikawa, H., and Yoshikawa, T. (2010). Increased expression of microRNA in the inflamed colonic mucosa of patients with active ulcerative colitis. *J Gastroenterol Hepatol* 25 Suppl 1**,** S129-133.

Tan, K.S., Armugam, A., Sepramaniam, S., Lim, K.Y., Setyowati, K.D., Wang, C.W., and Jeyaseelan, K. (2009). Expression profile of MicroRNAs in young stroke patients. *PLoS One* 4**,** e7689.

Tanaka, K., Miyata, H., Yamasaki, M., Sugimura, K., Takahashi, T., Kurokawa, Y., Nakajima, K., Takiguchi, S., Mori, M., and Doki, Y. (2013). Circulating miR-200c Levels Significantly Predict Response to Chemotherapy and Prognosis of Patients Undergoing Neoadjuvant Chemotherapy for Esophageal Cancer. *Ann Surg Oncol* 20 Suppl 3**,** 607-615.

Te, J.L., Dozmorov, I.M., Guthridge, J.M., Nguyen, K.L., Cavett, J.W., Kelly, J.A., Bruner, G.R., Harley, J.B., and Ojwang, J.O. (2010). Identification of unique microRNA signature associated with lupus nephritis. *PLoS One* 5**,** e10344.

Thorns, C., Kuba, J., Bernard, V., Senft, A., Szymczak, S., Feller, A.C., and Bernd, H.W. (2012). Deregulation of a distinct set of microRNAs is associated with transformation of gastritis into MALT lymphoma. *Virchows Arch* 460**,** 371-377.

Tokarz, P., and Blasiak, J. (2012). The role of microRNA in metastatic colorectal cancer and its significance in cancer prognosis and treatment. *Acta Biochim Pol* 59**,** 467-474.

Tombol, Z., Eder, K., Kovacs, A., Szabo, P.M., Kulka, J., Liko, I., Zalatnai, A., Racz, G., Toth, M., Patocs, A., Falus, A., Racz, K., and Igaz, P. (2010). MicroRNA expression profiling in benign (sporadic and hereditary) and recurring adrenal pheochromocytomas. *Mod Pathol* 23**,** 1583-1595.

Torres, A., Torres, K., Pesci, A., Ceccaroni, M., Paszkowski, T., Cassandrini, P., Zamboni, G., and Maciejewski, R. (2013). Diagnostic and prognostic significance of miRNA signatures in tissues and plasma of endometrioid endometrial carcinoma patients. *Int J Cancer* 132**,** 1633-1645.

Tu, H.F., Liu, C.J., Chang, C.L., Wang, P.W., Kao, S.Y., Yang, C.C., Yu, E.H., Lin, S.C., and Chang, K.W. (2012). The association between genetic polymorphism and the processing efficiency of miR-149 affects the prognosis of patients with head and neck squamous cell carcinoma. *PLoS One* 7**,** e51606.

Ueda, T., Volinia, S., Okumura, H., Shimizu, M., Taccioli, C., Rossi, S., Alder, H., Liu, C.G., Oue, N., Yasui, W., Yoshida, K., Sasaki, H., Nomura, S., Seto, Y., Kaminishi, M., Calin, G.A., and Croce, C.M. (2010). Relation between microRNA expression and progression and prognosis of gastric cancer: a microRNA expression analysis. *Lancet Oncol* 11**,** 136-146.

Vaishnavi, V., Manikandan, M., Tiwary, B.K., and Munirajan, A.K. (2013). Insights on the functional impact of microRNAs present in autism-associated copy number variants. *PLoS One* 8**,** e56781.

Valiyaveettil, M., Alamneh, Y.A., Miller, S.A., Hammamieh, R., Arun, P., Wang, Y., Wei, Y., Oguntayo, S., Long, J.B., and Nambiar, M.P. (2013). Modulation of cholinergic pathways and inflammatory mediators in blast-induced traumatic brain injury. *Chem Biol Interact* 203**,** 371-375.

Valladares-Ayerbes, M., Reboredo, M., Medina-Villaamil, V., Iglesias-Diaz, P., Lorenzo-Patino, M.J., Haz, M., Santamarina, I., Blanco, M., Fernandez-Tajes, J., Quindos, M., Carral, A., Figueroa, A., Anton-Aparicio, L.M., and Calvo, L. (2012). Circulating miR-200c as a diagnostic and prognostic biomarker for gastric cancer. *J Transl Med* 10**,** 186.

Van Kempen, L.C., Van Den Hurk, K., Lazar, V., Michiels, S., Winnepenninckx, V., Stas, M., Spatz, A., and Van Den Oord, J.J. (2012). Loss of microRNA-200a and c, and microRNA-203 expression at the invasive front of primary cutaneous melanoma is associated with increased thickness and disease progression. *Virchows Arch* 461**,** 441-448.

Van Pottelberge, G.R., Mestdagh, P., Bracke, K.R., Thas, O., Van Durme, Y.M., Joos, G.F., Vandesompele, J., and Brusselle, G.G. (2011). MicroRNA expression in induced sputum of smokers and patients with chronic obstructive pulmonary disease. *Am J Respir Crit Care Med* 183**,** 898-906.

Van Rooij, E., Sutherland, L.B., Thatcher, J.E., Dimaio, J.M., Naseem, R.H., Marshall, W.S., Hill, J.A., and Olson, E.N. (2008). Dysregulation of microRNAs after myocardial infarction reveals a role of miR-29 in cardiac fibrosis. *Proc Natl Acad Sci U S A* 105**,** 13027-13032.

Varnholt, H., Drebber, U., Schulze, F., Wedemeyer, I., Schirmacher, P., Dienes, H.P., and Odenthal, M. (2008). MicroRNA gene expression profile of hepatitis C virus-associated hepatocellular carcinoma. *Hepatology* 47**,** 1223-1232.

Vazquez, I., Maicas, M., Marcotegui, N., Conchillo, A., Guruceaga, E., Roman-Gomez, J., Calasanz, M.J., Agirre, X., Prosper, F., and Odero, M.D. (2010). Silencing of hsa-miR-124 by EVI1 in cell lines and patients with acute myeloid leukemia. *Proc Natl Acad Sci U S A* 107**,** E167-168; author reply E169-170.

Villa, C., Fenoglio, C., De Riz, M., Clerici, F., Marcone, A., Benussi, L., Ghidoni, R., Gallone, S., Cortini, F., Serpente, M., Cantoni, C., Fumagalli, G., Martinelli Boneschi, F., Cappa, S., Binetti, G., Franceschi, M., Rainero, I., Giordana, M.T., Mariani, C., Bresolin, N., Scarpini, E., and Galimberti, D. (2011). Role of hnRNP-A1 and miR-590-3p in neuronal death: genetics and expression analysis in patients with Alzheimer disease and frontotemporal lobar degeneration. *Rejuvenation Res* 14**,** 275-281.

Visone, R., Pallante, P., Vecchione, A., Cirombella, R., Ferracin, M., Ferraro, A., Volinia, S., Coluzzi, S., Leone, V., Borbone, E., Liu, C.G., Petrocca, F., Troncone, G., Calin, G.A., Scarpa, A., Colato, C., Tallini, G., Santoro, M., Croce, C.M., and Fusco, A. (2007). Specific microRNAs are downregulated in human thyroid anaplastic carcinomas. *Oncogene* 26**,** 7590-7595.

Voellenkle, C., Van Rooij, J., Cappuzzello, C., Greco, S., Arcelli, D., Di Vito, L., Melillo, G., Rigolini, R., Costa, E., Crea, F., Capogrossi, M.C., Napolitano, M., and Martelli, F. (2010). MicroRNA signatures in peripheral blood mononuclear cells of chronic heart failure patients. *Physiol Genomics* 42**,** 420-426.

Volinia, S., and Croce, C.M. (2013). Prognostic microRNA/mRNA signature from the integrated analysis of patients with invasive breast cancer. *Proc Natl Acad Sci U S A* 110**,** 7413-7417.

Vriens, M.R., Weng, J., Suh, I., Huynh, N., Guerrero, M.A., Shen, W.T., Duh, Q.Y., Clark, O.H., and Kebebew, E. (2012). MicroRNA expression profiling is a potential diagnostic tool for thyroid cancer. *Cancer* 118**,** 3426-3432.

Walter, B.A., Valera, V.A., Pinto, P.A., and Merino, M.J. (2013). Comprehensive microRNA Profiling of Prostate Cancer. *J Cancer* 4**,** 350-357.

Wang, F., Ma, Y.L., Zhang, P., Shen, T.Y., Shi, C.Z., Yang, Y.Z., Moyer, M.P., Zhang, H.Z., Chen, H.Q., Liang, Y., and Qin, H.L. (2013a). SP1 mediates the link between methylation of the tumour suppressor miR-149 and outcome in colorectal cancer. *J Pathol* 229**,** 12-24.

Wang, G., Chan, E.S., Kwan, B.C., Li, P.K., Yip, S.K., Szeto, C.C., and Ng, C.F. (2012a). Expression of microRNAs in the urine of patients with bladder cancer. *Clin Genitourin Cancer* 10**,** 106-113.

Wang, G., Kwan, B.C., Lai, F.M., Choi, P.C., Chow, K.M., Li, P.K., and Szeto, C.C. (2010). Intrarenal expression of miRNAs in patients with hypertensive nephrosclerosis. *Am J Hypertens* 23**,** 78-84.

Wang, G., Mao, W., Zheng, S., and Ye, J. (2009). Epidermal growth factor receptor-regulated miR-125a-5p--a metastatic inhibitor of lung cancer. *FEBS J* 276**,** 5571-5578.

Wang, G., Tam, L.S., Li, E.K., Kwan, B.C., Chow, K.M., Luk, C.C., Li, P.K., and Szeto, C.C. (2011a). Serum and urinary free microRNA level in patients with systemic lupus erythematosus. *Lupus* 20**,** 493-500.

Wang, H., Peng, W., Ouyang, X., Li, W., and Dai, Y. (2012b). Circulating microRNAs as candidate biomarkers in patients with systemic lupus erythematosus. *Transl Res* 160**,** 198-206.

Wang, H., Zhang, P., Chen, W., Feng, D., Jia, Y., and Xie, L. (2012c). Serum microRNA signatures identified by Solexa sequencing predict sepsis patients' mortality: a prospective observational study. *PLoS One* 7**,** e38885.

Wang, H.J., Guo, Y.Q., Tan, G., Dong, L., Cheng, L., Li, K.J., Wang, Z.Y., and Luo, H.F. (2013b). miR-125b regulates side population in breast cancer and confers a chemoresistant phenotype. *J Cell Biochem* 114**,** 2248-2257.

Wang, H.J., Zhang, P.J., Chen, W.J., Feng, D., Jia, Y.H., and Xie, L.X. (2012d). Four serum microRNAs identified as diagnostic biomarkers of sepsis. *J Trauma Acute Care Surg* 73**,** 850-854.

Wang, J., Huang, W., Xu, R., Nie, Y., Cao, X., Meng, J., Xu, X., Hu, S., and Zheng, Z. (2012e). MicroRNA-24 regulates cardiac fibrosis after myocardial infarction. *J Cell Mol Med* 16**,** 2150-2160.

Wang, J., Zhao, H., Tang, D., Wu, J., Yao, G., and Zhang, Q. (2013c). Overexpressions of MicroRNA-9 and MicroRNA-200c in Human Breast Cancers Are Associated with Lymph Node Metastasis. *Cancer Biother Radiopharm*.

Wang, M., Li, C., Nie, H., Lv, X., Qu, Y., Yu, B., Su, L., Li, J., Chen, X., Ju, J., Yu, Y., Yan, M., Gu, Q., Zhu, Z., and Liu, B. (2012f). Down-regulated miR-625 suppresses invasion and metastasis of gastric cancer by targeting ILK. *FEBS Lett* 586**,** 2382-2388.

Wang, M., Zhang, P., Li, Y., Liu, G., Zhou, B., Zhan, L., Zhou, Z., and Sun, X. (2012g). The quantitative analysis by stem-loop real-time PCR revealed the microRNA-34a, microRNA-155 and microRNA-200c overexpression in human colorectal cancer. *Med Oncol* 29**,** 3113-3118.

Wang, P., Chen, L., Zhang, J., Chen, H., Fan, J., Wang, K., Luo, J., Chen, Z., Meng, Z., and Liu, L. (2013d). Methylation-mediated silencing of the miR-124 genes facilitates pancreatic cancer progression and metastasis by targeting Rac1. *Oncogene*.

Wang, Q., Huang, Z., Ni, S., Xiao, X., Xu, Q., Wang, L., Huang, D., Tan, C., Sheng, W., and Du, X. (2012h). Plasma miR-601 and miR-760 are novel biomarkers for the early detection of colorectal cancer. *PLoS One* 7**,** e44398.

Wang, S., Xiang, J., Li, Z., Lu, S., Hu, J., Gao, X., Yu, L., Wang, L., Wang, J., Wu, Y., Chen, Z., and Zhu, H. (2013e). A plasma microRNA panel for early detection of colorectal cancer. *Int J Cancer*.

Wang, W., Corrigan-Cummins, M., Hudson, J., Maric, I., Simakova, O., Neelapu, S.S., Kwak, L.W., Janik, J.E., Gause, B., Jaffe, E.S., and Calvo, K.R. (2012i). MicroRNA profiling of follicular lymphoma identifies microRNAs related to cell proliferation and tumor response. *Haematologica* 97**,** 586-594.

Wang, W., Zhao, L.J., Tan, Y.X., Ren, H., and Qi, Z.T. (2012j). Identification of deregulated miRNAs and their targets in hepatitis B virus-associated hepatocellular carcinoma. *World J Gastroenterol* 18**,** 5442-5453.

Wang, W., Zhao, L.J., Tan, Y.X., Ren, H., and Qi, Z.T. (2012k). MiR-138 induces cell cycle arrest by targeting cyclin D3 in hepatocellular carcinoma. *Carcinogenesis* 33**,** 1113-1120.

Wang, W.X., Huang, Q., Hu, Y., Stromberg, A.J., and Nelson, P.T. (2011b). Patterns of microRNA expression in normal and early Alzheimer's disease human temporal cortex: white matter versus gray matter. *Acta Neuropathol* 121**,** 193-205.

Wang, Y., Zheng, X., Zhang, Z., Zhou, J., Zhao, G., Yang, J., Xia, L., Wang, R., Cai, X., Hu, H., Zhu, C., Nie, Y., Wu, K., Zhang, D., and Fan, D. (2012l). MicroRNA-149 inhibits proliferation and cell cycle progression through the targeting of ZBTB2 in human gastric cancer. *PLoS One* 7**,** e41693.

Wee, E.J., Peters, K., Nair, S.S., Hulf, T., Stein, S., Wagner, S., Bailey, P., Lee, S.Y., Qu, W.J., Brewster, B., French, J.D., Dobrovic, A., Francis, G.D., Clark, S.J., and Brown, M.A. (2012). Mapping the regulatory sequences controlling 93 breast cancer-associated miRNA genes leads to the identification of two functional promoters of the Hsa-mir-200b cluster, methylation of which is associated with metastasis or hormone receptor status in advanced breast cancer. *Oncogene* 31**,** 4182-4195.

Wei, X., Tan, C., Tang, C., Ren, G., Xiang, T., Qiu, Z., Liu, R., and Wu, Z. (2013). Epigenetic repression of miR-132 expression by the hepatitis B virus x protein in hepatitis B virus-related hepatocellular carcinoma. *Cell Signal* 25**,** 1037-1043.

Weng, L., Wu, X., Gao, H., Mu, B., Li, X., Wang, J.H., Guo, C., Jin, J.M., Chen, Z., Covarrubias, M., Yuan, Y.C., Weiss, L.M., and Wu, H. (2010). MicroRNA profiling of clear cell renal cell carcinoma by whole-genome small RNA deep sequencing of paired frozen and formalin-fixed, paraffin-embedded tissue specimens. *J Pathol* 222**,** 41-51.

White, N.M., Bao, T.T., Grigull, J., Youssef, Y.M., Girgis, A., Diamandis, M., Fatoohi, E., Metias, M., Honey, R.J., Stewart, R., Pace, K.T., Bjarnason, G.A., and Yousef, G.M. (2011). miRNA profiling for clear cell renal cell carcinoma: biomarker discovery and identification of potential controls and consequences of miRNA dysregulation. *J Urol* 186**,** 1077-1083.

White, N.M., Bui, A., Mejia-Guerrero, S., Chao, J., Soosaipillai, A., Youssef, Y., Mankaruos, M., Honey, R.J., Stewart, R., Pace, K.T., Sugar, L., Diamandis, E.P., Dore, J., and Yousef, G.M. (2010). Dysregulation of kallikrein-related peptidases in renal cell carcinoma: potential targets of miRNAs. *Biol Chem* 391**,** 411-423.

Willemen, H.L., Huo, X.J., Mao-Ying, Q.L., Zijlstra, J., Heijnen, C.J., and Kavelaars, A. (2012). MicroRNA-124 as a novel treatment for persistent hyperalgesia. *J Neuroinflammation* 9**,** 143.

Wilting, S.M., Snijders, P.J., Verlaat, W., Jaspers, A., Van De Wiel, M.A., Van Wieringen, W.N., Meijer, G.A., Kenter, G.G., Yi, Y., Le Sage, C., Agami, R., Meijer, C.J., and Steenbergen, R.D. (2013). Altered microRNA expression associated with chromosomal changes contributes to cervical carcinogenesis. *Oncogene* 32**,** 106-116.

Wilting, S.M., Van Boerdonk, R.A., Henken, F.E., Meijer, C.J., Diosdado, B., Meijer, G.A., Le Sage, C., Agami, R., Snijders, P.J., and Steenbergen, R.D. (2010). Methylation-mediated silencing and tumour suppressive function of hsa-miR-124 in cervical cancer. *Mol Cancer* 9**,** 167.

Won, K.Y., Kim, Y.W., Kim, H.S., Lee, S.K., Jung, W.W., and Park, Y.K. (2013). MicroRNA-199b-5p is involved in the Notch signaling pathway in osteosarcoma. *Hum Pathol* 44**,** 1648-1655.

Wong, G., and Nass, R. (2012). miRNAs and their putative roles in the development and progression of Parkinson's disease. *Front Genet* 3**,** 315.

Wong, H.K., Veremeyko, T., Patel, N., Lemere, C.A., Walsh, D.M., Esau, C., Vanderburg, C., and Krichevsky, A.M. (2013). De-repression of FOXO3a death axis by microRNA-132 and -212 causes neuronal apoptosis in Alzheimer's disease. *Hum Mol Genet* 22**,** 3077-3092.

Wong, K.Y., So, C.C., Loong, F., Chung, L.P., Lam, W.W., Liang, R., Li, G.K., Jin, D.Y., and Chim, C.S. (2011). Epigenetic inactivation of the miR-124-1 in haematological malignancies. *PLoS One* 6**,** e19027.

Wong, T.S., Liu, X.B., Wong, B.Y., Ng, R.W., Yuen, A.P., and Wei, W.I. (2008). Mature miR-184 as Potential Oncogenic microRNA of Squamous Cell Carcinoma of Tongue. *Clin Cancer Res* 14**,** 2588-2592.

Wotschofsky, Z., Busch, J., Jung, M., Kempkensteffen, C., Weikert, S., Schaser, K.D., Melcher, I., Kilic, E., Miller, K., Kristiansen, G., Erbersdobler, A., and Jung, K. (2013). Diagnostic and prognostic potential of differentially expressed miRNAs between metastatic and non-metastatic renal cell carcinoma at the time of nephrectomy. *Clin Chim Acta* 416**,** 5-10.

Wszolek, M.F., Rieger-Christ, K.M., Kenney, P.A., Gould, J.J., Silva Neto, B., Lavoie, A.K., Logvinenko, T., Libertino, J.A., and Summerhayes, I.C. (2011). A MicroRNA expression profile defining the invasive bladder tumor phenotype. *Urol Oncol* 29**,** 794-801 e791.

Wu, C., Lin, H., Wang, Q., Chen, W., Luo, H., Chen, W., and Zhang, H. (2012a). Discrepant expression of microRNAs in transparent and cataractous human lenses. *Invest Ophthalmol Vis Sci* 53**,** 3906-3912.

Wu, J.H., Gao, Y., Ren, A.J., Zhao, S.H., Zhong, M., Peng, Y.J., Shen, W., Jing, M., and Liu, L. (2012b). Altered microRNA expression profiles in retinas with diabetic retinopathy. *Ophthalmic Res* 47**,** 195-201.

Wu, Q., Wang, C., Lu, Z., Guo, L., and Ge, Q. (2012c). Analysis of serum genome-wide microRNAs for breast cancer detection. *Clin Chim Acta* 413**,** 1058-1065.

Wu, W., Lin, Z., Zhuang, Z., and Liang, X. (2009). Expression profile of mammalian microRNAs in endometrioid adenocarcinoma. *Eur J Cancer Prev* 18**,** 50-55.

Wu, W., Qin, Y., Li, Z., Dong, J., Dai, J., Lu, C., Guo, X., Zhao, Y., Zhu, Y., Zhang, W., Hang, B., Sha, J., Shen, H., Xia, Y., Hu, Z., and Wang, X. (2013a). Genome-wide microRNA expression profiling in idiopathic non-obstructive azoospermia: significant up-regulation of miR-141, miR-429 and miR-7-1-3p. *Hum Reprod* 28**,** 1827-1836.

Wu, X., Liu, T., Fang, O., Leach, L.J., Hu, X., and Luo, Z. (2013b). miR-194 suppresses metastasis of non-small cell lung cancer through regulating expression of BMP1 and p27. *Oncogene*.

Wu, X., Weng, L., Li, X., Guo, C., Pal, S.K., Jin, J.M., Li, Y., Nelson, R.A., Mu, B., Onami, S.H., Wu, J.J., Ruel, N.H., Wilczynski, S.P., Gao, H., Covarrubias, M., Figlin, R.A., Weiss, L.M., and Wu, H. (2012d). Identification of a 4-microRNA signature for clear cell renal cell carcinoma metastasis and prognosis. *PLoS One* 7**,** e35661.

Xia, J., Wu, Z., Yu, C., He, W., Zheng, H., He, Y., Jian, W., Chen, L., Zhang, L., and Li, W. (2012). miR-124 inhibits cell proliferation in gastric cancer through down-regulation of SPHK1. *J Pathol* 227**,** 470-480.

Xiao, W., Bao, Z.X., Zhang, C.Y., Zhang, X.Y., Shi, L.J., Zhou, Z.T., and Jiang, W.W. (2012). Upregulation of miR-31* is negatively associated with recurrent/newly formed oral leukoplakia. *PLoS One* 7**,** e38648.

Xie, Y.F., Shu, R., Jiang, S.Y., Liu, D.L., and Zhang, X.L. (2011). Comparison of microRNA profiles of human periodontal diseased and healthy gingival tissues. *Int J Oral Sci* 3**,** 125-134.

Xu, N., Brodin, P., Wei, T., Meisgen, F., Eidsmo, L., Nagy, N., Kemeny, L., Stahle, M., Sonkoly, E., and Pivarcsi, A. (2011). MiR-125b, a microRNA downregulated in psoriasis, modulates keratinocyte proliferation by targeting FGFR2. *J Invest Dermatol* 131**,** 1521-1529.

Xu, Y., Brenn, T., Brown, E.R., Doherty, V., and Melton, D.W. (2012). Differential expression of microRNAs during melanoma progression: miR-200c, miR-205 and miR-211 are downregulated in melanoma and act as tumour suppressors. *Br J Cancer* 106**,** 553-561.

Yan, L.X., Huang, X.F., Shao, Q., Huang, M.Y., Deng, L., Wu, Q.L., Zeng, Y.X., and Shao, J.Y. (2008). MicroRNA miR-21 overexpression in human breast cancer is associated with advanced clinical stage, lymph node metastasis and patient poor prognosis. *RNA* 14**,** 2348-2360.

Yan, W., Zhang, W., Sun, L., Liu, Y., You, G., Wang, Y., Kang, C., You, Y., and Jiang, T. (2011). Identification of MMP-9 specific microRNA expression profile as potential targets of anti-invasion therapy in glioblastoma multiforme. *Brain Res* 1411**,** 108-115.

Yan, X., Huang, Y., Zhao, J.X., Rogers, C.J., Zhu, M.J., Ford, S.P., Nathanielsz, P.W., and Du, M. (2013). Maternal obesity downregulates microRNA let-7g expression, a possible mechanism for enhanced adipogenesis during ovine fetal skeletal muscle development. *Int J Obes (Lond)* 37**,** 568-575.

Yang, J., Gao, T., Tang, J., Cai, H., Lin, L., and Fu, S. (2013). Loss of microRNA-132 predicts poor prognosis in patients with primary osteosarcoma. *Mol Cell Biochem* 381**,** 9-15.

Yang, S., Banerjee, S., De Freitas, A., Sanders, Y.Y., Ding, Q., Matalon, S., Thannickal, V.J., Abraham, E., and Liu, G. (2012). Participation of miR-200 in pulmonary fibrosis. *Am J Pathol* 180**,** 484-493.

Yang, Y., Li, X., Yang, Q., Wang, X., Zhou, Y., Jiang, T., Ma, Q., and Wang, Y.J. (2010). The role of microRNA in human lung squamous cell carcinoma. *Cancer Genet Cytogenet* 200**,** 127-133.

Yao, Y., Suo, A.L., Li, Z.F., Liu, L.Y., Tian, T., Ni, L., Zhang, W.G., Nan, K.J., Song, T.S., and Huang, C. (2009). MicroRNA profiling of human gastric cancer. *Mol Med Rep* 2**,** 963-970.

Yi, Z., Fu, Y., Ji, R., Li, R., and Guan, Z. (2012). Altered microRNA signatures in sputum of patients with active pulmonary tuberculosis. *PLoS One* 7**,** e43184.

Youssef, Y.M., White, N.M., Grigull, J., Krizova, A., Samy, C., Mejia-Guerrero, S., Evans, A., and Yousef, G.M. (2011). Accurate molecular classification of kidney cancer subtypes using microRNA signature. *Eur Urol* 59**,** 721-730.

Yu, F., Yao, H., Zhu, P., Zhang, X., Pan, Q., Gong, C., Huang, Y., Hu, X., Su, F., Lieberman, J., and Song, E. (2007). let-7 regulates self renewal and tumorigenicity of breast cancer cells. *Cell* 131**,** 1109-1123.

Yu, J., Li, A., Hong, S.M., Hruban, R.H., and Goggins, M. (2012). MicroRNA alterations of pancreatic intraepithelial neoplasias. *Clin Cancer Res* 18**,** 981-992.

Yu, J., Ohuchida, K., Mizumoto, K., Sato, N., Kayashima, T., Fujita, H., Nakata, K., and Tanaka, M. (2010). MicroRNA, hsa-miR-200c, is an independent prognostic factor in pancreatic cancer and its upregulation inhibits pancreatic cancer invasion but increases cell proliferation. *Mol Cancer* 9**,** 169.

Yu, Z., Ni, L., Chen, D., Zhang, Q., Su, Z., Wang, Y., Yu, W., Wu, X., Ye, J., Yang, S., Lai, Y., and Li, X. (2013). Identification of miR-7 as an oncogene in renal cell carcinoma. *J Mol Histol* 44**,** 669-677.

Yuan, M., Zhan, Q., Duan, X., Song, B., Zeng, S., Chen, X., Yang, Q., and Xia, J. (2013). A functional polymorphism at miR-491-5p binding site in the 3'-UTR of MMP-9 gene confers increased risk for atherosclerotic cerebral infarction in a Chinese population. *Atherosclerosis* 226**,** 447-452.

Yuxia, M., Zhennan, T., and Wei, Z. (2012). Circulating miR-125b is a novel biomarker for screening non-small-cell lung cancer and predicts poor prognosis. *J Cancer Res Clin Oncol* 138**,** 2045-2050.

Zahm, A.M., Hand, N.J., Boateng, L.A., and Friedman, J.R. (2012). Circulating microRNA is a biomarker of biliary atresia. *J Pediatr Gastroenterol Nutr* 55**,** 366-369.

Zampetaki, A., Kiechl, S., Drozdov, I., Willeit, P., Mayr, U., Prokopi, M., Mayr, A., Weger, S., Oberhollenzer, F., Bonora, E., Shah, A., Willeit, J., and Mayr, M. (2010). Plasma microRNA profiling reveals loss of endothelial miR-126 and other microRNAs in type 2 diabetes. *Circ Res* 107**,** 810-817.

Zeng, B., Li, Z., Chen, R., Guo, N., Zhou, J., Zhou, Q., Lin, Q., Cheng, D., Liao, Q., Zheng, L., and Gong, Y. (2012). Epigenetic regulation of miR-124 by hepatitis C virus core protein promotes migration and invasion of intrahepatic cholangiocarcinoma cells by targeting SMYD3. *FEBS Lett* 586**,** 3271-3278.

Zhang, H., Luo, X.Q., Feng, D.D., Zhang, X.J., Wu, J., Zheng, Y.S., Chen, X., Xu, L., and Chen, Y.Q. (2011a). Upregulation of microRNA-125b contributes to leukemogenesis and increases drug resistance in pediatric acute promyelocytic leukemia. *Mol Cancer* 10**,** 108.

Zhang, H.B., Li, R.C., Xu, M., Xu, S.M., Lai, Y.S., Wu, H.D., Xie, X.J., Gao, W., Ye, H., Zhang, Y.Y., Meng, X., and Wang, S.Q. (2013). Ultrastructural uncoupling between T-tubules and sarcoplasmic reticulum in human heart failure. *Cardiovasc Res* 98**,** 269-276.

Zhang, M.W., Jin, M.J., Yu, Y.X., Zhang, S.C., Liu, B., Jiang, X., Pan, Y.F., Li, Q.I., Ma, S.Y., and Chen, K. (2012a). Associations of lifestyle-related factors, hsa-miR-149 and hsa-miR-605 gene polymorphisms with gastrointestinal cancer risk. *Mol Carcinog* 51 Suppl 1**,** E21-31.

Zhang, S., Hao, J., Xie, F., Hu, X., Liu, C., Tong, J., Zhou, J., Wu, J., and Shao, C. (2011b). Downregulation of miR-132 by promoter methylation contributes to pancreatic cancer development. *Carcinogenesis* 32**,** 1183-1189.

Zhang, Y., Li, M., Wang, H., Fisher, W.E., Lin, P.H., Yao, Q., and Chen, C. (2009). Profiling of 95 microRNAs in pancreatic cancer cell lines and surgical specimens by real-time PCR analysis. *World J Surg* 33**,** 698-709.

Zhang, Y.K., Zhu, W.Y., He, J.Y., Chen, D.D., Huang, Y.Y., Le, H.B., and Liu, X.G. (2012b). miRNAs expression profiling to distinguish lung squamous-cell carcinoma from adenocarcinoma subtypes. *J Cancer Res Clin Oncol* 138**,** 1641-1650.

Zhang, Z., Chang, H., Li, Y., Zhang, T., Zou, J., Zheng, X., and Wu, J. (2010). MicroRNAs: potential regulators involved in human anencephaly. *Int J Biochem Cell Biol* 42**,** 367-374.

Zhao, B.S., Liu, S.G., Wang, T.Y., Ji, Y.H., Qi, B., Tao, Y.P., Li, H.C., and Wu, X.N. (2013a). Screening of microRNA in patients with esophageal cancer at same tumor node metastasis stage with different prognoses. *Asian Pac J Cancer Prev* 14**,** 139-143.

Zhao, C., Dong, J., Jiang, T., Shi, Z., Yu, B., Zhu, Y., Chen, D., Xu, J., Huo, R., Dai, J., Xia, Y., Pan, S., Hu, Z., and Sha, J. (2011). Early second-trimester serum miRNA profiling predicts gestational diabetes mellitus. *PLoS One* 6**,** e23925.

Zhao, J.J., Yang, J., Lin, J., Yao, N., Zhu, Y., Zheng, J., Xu, J., Cheng, J.Q., Lin, J.Y., and Ma, X. (2009). Identification of miRNAs associated with tumorigenesis of retinoblastoma by miRNA microarray analysis. *Childs Nerv Syst* 25**,** 13-20.

Zhao, L., Sun, Y., Hou, Y., Peng, Q., Wang, L., Luo, H., Tang, X., Zeng, Z., and Liu, M. (2012). MiRNA expression analysis of cancer-associated fibroblasts and normal fibroblasts in breast cancer. *Int J Biochem Cell Biol* 44**,** 2051-2059.

Zhao, W.H., Wu, S.Q., and Zhang, Y.D. (2013b). Downregulation of miR-124 promotes the growth and invasiveness of glioblastoma cells involving upregulation of PPP1R13L. *Int J Mol Med* 32**,** 101-107.

Zhao, X., Dou, W., He, L., Liang, S., Tie, J., Liu, C., Li, T., Lu, Y., Mo, P., Shi, Y., Wu, K., Nie, Y., and Fan, D. (2013c). MicroRNA-7 functions as an anti-metastatic microRNA in gastric cancer by targeting insulin-like growth factor-1 receptor. *Oncogene* 32**,** 1363-1372.

Zheng, H.W., Wang, Y.L., Lin, J.X., Li, N., Zhao, X.Q., Liu, G.F., Liu, L.P., Jiao, Y., Gu, W.K., Wang, D.Z., and Wang, Y.J. (2012). Circulating MicroRNAs as potential risk biomarkers for hematoma enlargement after intracerebral hemorrhage. *CNS Neurosci Ther* 18**,** 1003-1011.

Zhi, F., Cao, X., Xie, X., Wang, B., Dong, W., Gu, W., Ling, Y., Wang, R., Yang, Y., and Liu, Y. (2013). Identification of circulating microRNAs as potential biomarkers for detecting acute myeloid leukemia. *PLoS One* 8**,** e56718.

Zhi, F., Chen, X., Wang, S., Xia, X., Shi, Y., Guan, W., Shao, N., Qu, H., Yang, C., Zhang, Y., Wang, Q., Wang, R., Zen, K., Zhang, C.Y., Zhang, J., and Yang, Y. (2010). The use of hsa-miR-21, hsa-miR-181b and hsa-miR-106a as prognostic indicators of astrocytoma. *Eur J Cancer* 46**,** 1640-1649.

Zhou, L., Chen, J., Li, Z., Li, X., Hu, X., Huang, Y., Zhao, X., Liang, C., Wang, Y., Sun, L., Shi, M., Xu, X., Shen, F., Chen, M., Han, Z., Peng, Z., Zhai, Q., Chen, J., Zhang, Z., Yang, R., Ye, J., Guan, Z., Yang, H., Gui, Y., Wang, J., Cai, Z., and Zhang, X. (2010). Integrated profiling of microRNAs and mRNAs: microRNAs located on Xq27.3 associate with clear cell renal cell carcinoma. *PLoS One* 5**,** e15224.

Zhu, H., and Fan, G.C. (2012). Role of microRNAs in the reperfused myocardium towards post-infarct remodelling. *Cardiovasc Res* 94**,** 284-292.

Zongaro, S., Hukema, R., D'antoni, S., Davidovic, L., Barbry, P., Catania, M.V., Willemsen, R., Mari, B., and Bardoni, B. (2013). The 3' UTR of FMR1 mRNA is a target of miR-101, miR-129-5p and miR-221: implications for the molecular pathology of FXTAS at the synapse. *Hum Mol Genet* 22**,** 1971-1982.
